# Community-Level Pharmaceutical Interventions to Reduce the Risks of Polypharmacy in the Elderly: Overview of Systematic Reviews and Economic Evaluations

**DOI:** 10.3389/fphar.2019.00302

**Published:** 2019-04-02

**Authors:** Orenzio Soler, Jorge Otávio Maia Barreto

**Affiliations:** ^1^School of Pharmacy, Health Science Institute, Federal University of Pará, Belém, Brazil; ^2^Fiocruz School of Government, Fiocruz Brasília, Osvaldo Cruz Foundation, Brasília, Brazil

**Keywords:** elderly, polypharmacy, pharmaceutical interventions, overview, systematic review

## Abstract

**Background:** Patients over 65 years of age taking multiple medications face several risks, and pharmaceutical interventions can be useful to improve quality of care and reduce those risks. However, there is still no consensus on the effectiveness of these interventions aimed at promoting changes in clinical, epidemiological, economic, and humanistic outcomes for various service delivery, organizational, financial, and implementation-based interventions. The objective of this overview of systematic reviews was to summarize evidence on the effectiveness of community-level pharmaceutical interventions to reduce the risks associated with polypharmacy in the population over 65 years of age.

**Method:** This overview used a previously described protocol to search for systematic review articles, with and without meta-analysis, and economic evaluations, without any language or time restrictions, including articles published up to May 2018. The following databases were searched: the Cochrane Library, Epistemonikos, Health Evidence, Health Systems Evidence, Virtual Health Library, and Google Scholar. The basic search terms used were “elderly,” “polypharmacy,” and “pharmaceutical interventions.” The findings for outcomes of interest were categorized using a taxonomy for health policies and systems. Equity-related questions were also investigated. The studies were evaluated for methodological quality and produced a narrative synthesis.

**Results:** A total of 642 records were retrieved: 50 from Health Evidence, 197 from Epistemonikos, 194 from Cochrane, 116 from Health Systems Evidence, and 85 from the Virtual Health Library. Of these, 16 articles were selected: 1 overview of systematic reviews, 12 systematic reviews, and 3 economic evaluations. There is evidence of improvement in clinical, epidemiological, humanistic, and economic outcomes for various types of community-level pharmaceutical interventions, but differences in observed outcomes may be due to study designs, primary study sample sizes, risk of bias, difficulty in aggregating data, heterogeneity of indicators and quality of evidence included in the systematic reviews that were assessed. It is necessary to optimize the methodological designs of future primary and secondary studies.

**Conclusion:** Community-level pharmaceutical interventions can improve various clinical, epidemiological, humanistic and economic outcomes and potentially reduce risks associated with polypharmacy in the elderly population.

## Introduction

It is estimated that 21% of the world's population will be over 65 years of age by the year 2050. The elderly have complex health needs as they often have multiple comorbidities. An estimated 30% of elderly persons are prescribed 5 to 12 medications (United Nations, 2017). The elderly not only use more medications but also experience physiological changes, i.e., pharmacokinetic and pharmacodynamic changes, that increase the risk of adverse events. Between 10 and 30% of hospitalizations in this population are consequences of drug-related complications, which are potentially avoidable through adequate management (World Health Organization, 2015). The provision of care to this population represents one of the greatest challenges for health systems worldwide.

Community-level care for the elderly can be provided in various types of facilities, including community pharmacies. The terminology used to describe care units for the elderly differs around the world: hospices, long-term care facilities, nursing homes, skilled nursing facilities, and assisted living facilities (Pinto and Von-Simson, 2012). These types of facilities vary with regard to their infrastructure, the profile of the professionals employed, and the type of care offered (partial or full and/or individual or collective).

Elderly persons, particularly those residing in nursing homes, are susceptible to polypharmacy. Polypharmacy is defined as the prescription of multiple drugs to an individual (Duerden et al., 2013). The negative consequences of polypharmacy include prescription errors (PE), potentially inappropriate prescription (PIP), and potentially inappropriate medication (PIM), which can lead to drug-related problems (DRP) and/or drug-related negative outcomes (DNO) such as adverse drug events (ADEs) and/or adverse drug reactions (ADRs). The prevalence of polypharmacy in the elderly is high, although it varies widely depending on the definitions used, the facility type and the geographical location (Santos et al., 2007; Brasil Ministério da Saúde, 2014; Leelakanok et al., 2017).

The differences between “adequate polypharmacy” and “inadequate polypharmacy” are now recognized. “Adequate polypharmacy” occurs when multiple drugs are prescribed to an individual with multiple morbidities, in an evidence-based manner; i.e., the combination of prescribed medications will ensure a good quality of life, improve longevity, and minimize drug toxicity. “Inadequate polypharmacy” occurs when multiple medications are inappropriately prescribed, beyond the clinical needs; that is, when the intended benefit with the drug is not achieved, leading to unnecessary risks and negative health outcomes (Duerden et al., 2013).

Improving the quality of medication prescribing for the elderly also involves reducing the irrational use of medications, leading to better health outcomes. To address this challenge, frameworks for the evaluation of key factors related to the occurrence of inappropriate prescriptions as well as interventions to improve this professional conduct have been developed.

According to the Canadian Agency for Drugs and Technologies in Health (CADTH), there are several types of interventions targeted at various levels and components of health systems (Higgins and Green, 2011):

- Professional: (i) Interventions targeted at professionals to improve their prescribing practices; (ii) Interventions targeted at consumers to improve the use of medications.
- Organizational: Interventions that involve a change in the structure or delivery of health care.
- Financial: Interventions that focus on professional reimbursement, incentives, and penalties.
- Regulatory: Interventions that aim to change the provision of health services through regulatory frameworks.

These interventions require health professionals to analyze the pharmacotherapeutic strategy established for a patient. This is a continuous process that identifies and solves DRP and/or DNO based on need, efficacy and safety, with the goal of increasing effectiveness and decreasing the risks of pharmacotherapy. Examples include therapeutic strategy-related interventions, interventions related to the quantity of drugs and health education interventions (Brasil Ministério da Saúde, 2014). It is hypothesized, although there is no consensus, that professional, organizational, regulatory and financial interventions targeted at prescribers and consumers can be effective for improving the prescription and rational use of medications.

### Goal

This overview investigated the available evidence on the effects of community-level pharmaceutical interventions to reduce the risks associated with polypharmacy in the elderly population over 65 years of age.

## Methods

### Search Strategy

This overview covered studies published in the following databases: Cochrane Library, Epistemonikos, Health Evidence, Health Systems Evidence, Virtual Health Library (Portuguese acronym: BVS), and Google Scholar. There was no language or time restriction, including articles published up to May 2018. Systematic reviews, with or without meta-analysis, and economic evaluations were included. The search strategy included medical subject headings (MeSH) and health sciences descriptors (DeCS), using the keywords “Elderly,” “Polypharmacy,” and “Pharmaceutical intervention.” The search was adapted to the various electronic databases. Details of the search strategies are provided in Supplementary Material 1.

This study addressed the following question: Which community-level pharmaceutical interventions reduce the risks associated with polypharmacy in the elderly population over 65 years of age? In accordance with the PICO guidelines (Santos et al., 2007), studies with the following characteristics were included: Population (P): Individuals over 65 years of age; Intervention (I): Pharmaceutical interventions (pharmaceutical care); Control (C): No pharmaceutical intervention or any other intervention; and Outcome (O): Clinical, epidemiological, humanistic, and economic outcomes.

Studies focused on other age groups, such as adolescents and adults aged between 18 and 64 years, were excluded, along with studies that addressed interventions at other levels of care. The interventions of interest were those focused on identifying and solving problems related to polypharmacy, pharmaceutical care and reduction of the risks of medication use, at the community level.

### Review Process

#### Data Selection, Categorization, and Extraction

**Figure 1:**
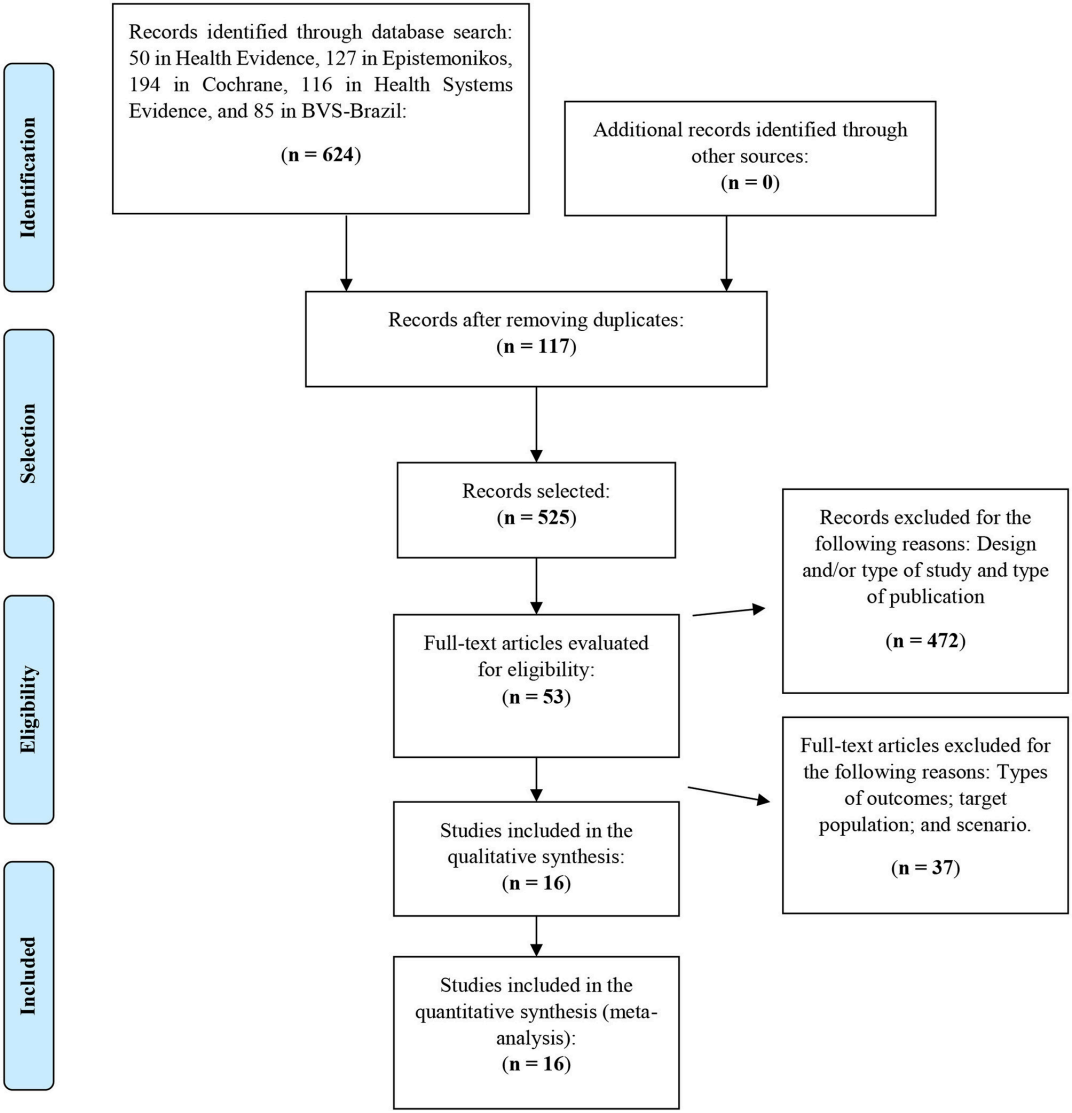
Flowchart of the selection process of the articles included in the review. Adapted from Moher et al. (2009). Complementary information: www.prisma-statement.org.

The identification and selection of studies followed the Cochrane Collaboration methods for systematic reviews (Higgins and Green, 2011). The retrieved studies were imported into the Rayyan QCRI (Ouzzani et al., 2016) online platform, and the references from the included and excluded studies were imported into the Mendeley reference manager (Mendeley et al., 2017). The titles and abstracts of the retrieved studies were independently selected by two reviewers (OS; JB). All disagreements were resolved by consensus among the reviewers. The selection process was documented and is presented in the flowchart adapted from the Preferred Reporting Items for Systematic Reviews and Meta-Analyzes (PRISMA) guidelines (Moher et al., 2009) (Figure 1).

For the categorization of pharmaceutical interventions, the taxonomy proposed by the CADTH (Canadian Agency for Drugs Technologies in Health., 2018) and the Cochrane Effective Practice and Organization of Care (EPOC) taxonomy (Khalil et al., 2017) were used to classify interventions as professional, organizational, financial, regulatory or multifaceted. The outcomes of these interventions were compared to the outcomes of interventions by other professionals, outcomes with no pharmaceutical intervention or outcomes with any other community-level intervention. As outcome criterion, clinical and humanistic outcomes, including access to services (output), as well as epidemiological and economic outcomes were used. Other definitions and concepts are available in Supplementary Material 2.

An extraction form was used to collect the data of interest: article title, authors, journal, year of publication, last year of research, objectives, methods, statistics, risk of bias, main results, gaps, limitations, recommendations, equity analysis, quality assessment, conflicts of interest, and unanswered questions.

### Excluded Articles

In total, 642 records were retrieved: 50 from Health Evidence, 197 from Epistemonikos, 194 from Cochrane, 116 from Health Systems Evidence, and 85 from the Virtual Health Library, with no articles retrieved from Google Scholar. Of these, 117 duplicates were removed, leaving 525 records. The titles and abstracts of the eligible studies were independently assessed by two reviewers (OS; JB). During the screening, 472 articles were excluded due to inadequacies regarding outcomes, target population, design and/or type of study and type of publication. A total of 53 articles were preselected based on the inclusion criteria. After reading the full text of the articles, 27 were excluded because of the scenarios, intervention types and outcomes. In the end, 16 articles were selected for this overview. The list of excluded articles is available in Supplementary Material 3.

### Data Synthesis

A narrative-descriptive synthesis was prepared. This synthesis describes the interventions and evidence found, including the main findings relevant to the aims of this overview.

#### Quality Assessment of Included Studies

The quality assessment was performed independently for each study by two reviewers (OS; JB), and the results were compared. Disagreements were resolved by consensus. We used A MeaSurement Tool to Assess systematic Reviews (AMSTAR) (Shea et al., 2007) and the checklist for Assessment of Economic Evaluation Studies (AEES) (Silva et al., 2014) to evaluate the quality of systematic reviews. Individual evaluations are available in Supplementary Materials 4, 5.

### Equity Considerations

Equity aspects were considered for the included studies, in particular, design approaches and reporting of issues related to health inequities. We used the PROGRESS framework (National Collaborating Centre for Methods and Tools, 2015), which includes place of residence, race/ethnicity/culture/language, occupation, sex/gender, religion, education level, socioeconomic status, and social capital. The PROGRESS framework was used to identify whether there were approaches and/or issues related to inequities in the outcomes of interest, in order to develop research and/or adapt research evidence and inform the design of new interventions (O'Neill et al., 2014).

The protocol of this overview was previously registered in PROSPERO (Booth, 2013) under number CRD42018093788/2018 (Soler and Barreto, 2018).

## Results

### Profile and Characteristics of the Reviews

Sixteen articles met the inclusion criteria. The included studies were published between 2007 and 2017. They included 1 overview of systematic reviews, 12 systematic reviews, and 3 economic evaluations. The countries where the primary studies in these reviews were conducted include high-, middle-, and low-income countries. The characteristics of the included studies are provided in Supplementary Material 6 and the distribution of studies by country and continent in Supplementary Material 7.

### Categories of Interventions and Outcomes

The categorization of interventions and outcomes based on the adopted frameworks is shown in Tables 1, 2. Clinical, epidemiological and humanistic outcomes, including access to services, were categorized for professional, organizational, financial, regulatory, and multifaceted interventions.

**Table 1:** Map of evidence on interventions for clinical outcomes.

**Interventions**			**Clinical outcomes**						
			**Polypharmacy: medication errors**		**Adherence**	**Drug-related problems**			
			**Reduction of potentially inappropriate prescriptions (PIP)**	**Improvement of use of appropriate and safe medications**	**Improvement of adherence**	**Reduction of adverse drug events**	**Reduction of adverse drug reactions**	**Reduction of drug-drug interactions**	**Reduction of drug-related negative health Outcomes**
Professional interventions	Targeted at prescribers	Clinical case analysis and/or evaluation	(Kaur et al., 2009; Alldred et al., 2013; Olaniyan et al., 2015)	(Kaur et al., 2009; Alldred et al., 2013; Olaniyan et al., 2015)	(Kaur et al., 2009; Alldred et al., 2013; Olaniyan et al., 2015)	^*^	^*^	^*^	^*^
		Review of drug use	(Hajjar et al., 2007; Kaur et al., 2009; Mathumalar et al., 2011; Patterson et al., 2012; Cooper et al., 2015; Jórdan-Sánchez et al., 2015; Olaniyan et al., 2015; Khalil et al., 2017)	(Hajjar et al., 2007; Kaur et al., 2009; Mathumalar et al., 2011; Patterson et al., 2012; Cooper et al., 2015; Jórdan-Sánchez et al., 2015; Olaniyan et al., 2015; Khalil et al., 2017)	(Hajjar et al., 2007; Kaur et al., 2009; Mathumalar et al., 2011; Patterson et al., 2012; Cooper et al., 2015; Jórdan-Sánchez et al., 2015; Olaniyan et al., 2015; Khalil et al., 2017)	(Hajjar et al., 2007)	(Hajjar et al., 2007)	(Hajjar et al., 2007)	(Hajjar et al., 2007)
		Educational interventions for prescribers	(Hajjar et al., 2007; Kaur et al., 2009; Mathumalar et al., 2011; Olaniyan et al., 2015)	(Hajjar et al., 2007; Kaur et al., 2009; Mathumalar et al., 2011; Olaniyan et al., 2015)	(Hajjar et al., 2007; Kaur et al., 2009; Mathumalar et al., 2011; Olaniyan et al., 2015)	^*^	^*^	^*^	^*^
	Targeted at users and caregivers	Educational interventions for users and/or caregivers	(Kaur et al., 2009; Mathumalar et al., 2011; Olaniyan et al., 2015)	(Kaur et al., 2009; Mathumalar et al., 2011; Olaniyan et al., 2015)	(Kaur et al., 2009; Mathumalar et al., 2011; Olaniyan et al., 2015)	^*^	^*^	^*^	^*^
Organizational interventions	Use of information and communication technology	Active search for user data and information	(Olaniyan et al., 2015)	(Olaniyan et al., 2015)	(Olaniyan et al., 2015)	^*^	^*^	^*^	^*^
		Drug information services	(Kaur et al., 2009)	(Kaur et al., 2009)	(Kaur et al., 2009)	^*^	^*^	^*^	^*^
		Clinical decision-making support systems	(Kaur et al., 2009; Mathumalar et al., 2011; Cooper et al., 2015)	(Kaur et al., 2009; Mathumalar et al., 2011; Cooper et al., 2015)	(Kaur et al., 2009; Mathumalar et al., 2011; Cooper et al., 2015)	^*^	^*^	^*^	^*^
		Use of risk screening tools	(Patterson et al., 2012; Olaniyan et al., 2015)	(Patterson et al., 2012; Olaniyan et al., 2015)	(Patterson et al., 2012; Olaniyan et al., 2015)	^*^	^*^	^*^	^*^
	Provision of pharmaceutical care services		(Mathumalar et al., 2011; Patterson et al., 2012; Lee et al., 2013; Sáez-Benito et al., 2013; Cooper et al., 2015; Olaniyan et al., 2015; Babar et al., 2017)	(Mathumalar et al., 2011; Patterson et al., 2012; Lee et al., 2013; Sáez-Benito et al., 2013; Cooper et al., 2015; Olaniyan et al., 2015; Babar et al., 2017)	(Mathumalar et al., 2011; Patterson et al., 2012; Lee et al., 2013; Sáez-Benito et al., 2013; Cooper et al., 2015; Olaniyan et al., 2015; Babar et al., 2017)	(Lee et al., 2013)	(Lee et al., 2013)	(Lee et al., 2013)	(Lee et al., 2013)
	Technical management of medications		(Olaniyan et al., 2015)	(Olaniyan et al., 2015)	(Olaniyan et al., 2015)	^*^	^*^	^*^	^*^
	Financial interventions: incentive programs to change prescribing practices		^*^	^*^	^*^	^*^	^*^	^*^	^*^
Governmental Interventions	Regulatory interventions: governmental policies regulating prescriptions		(Kaur et al., 2009)	(Kaur et al., 2009)	(Kaur et al., 2009)	^*^	^*^	^*^	^*^
Multifaceted interventions			(Hajjar et al., 2007; Alldred et al., 2013; Lee et al., 2013; Cooper et al., 2015; Olaniyan et al., 2015)	(Hajjar et al., 2007; Alldred et al., 2013; Lee et al., 2013; Cooper et al., 2015; Olaniyan et al., 2015)	(Hajjar et al., 2007; Alldred et al., 2013; Lee et al., 2013; Cooper et al., 2015; Olaniyan et al., 2015)	Sáez-Benito et al., 2013	Sáez-Benito et al., 2013	Sáez-Benito et al., 2013	Sáez-Benito et al., 2013

*The references in parentheses indicate the studies that presented evidence for each intervention/outcome studied*.

* *Not studied and/or no evidence*.

*community-level pharmaceutical interventions to reduce the risks of polypharmacy in the elderly: overview of systematic reviews and economic evaluations*.

**Table 2:** Map of evidence on interventions for access to services, epidemiological, humanistic, and economic outcomes.

**Interventions**			**Access to services (output)**					**Epidemiological outcomes**		**Humanistic outcomes**		**Economic outcomes**
			**Reduction of outpatient visits**	**Reduction of house visits**	**Reduction of visits to urgent and emergency services**	**Reduction of hospitalizations**	**Reduction of hospitalization time**	**Reductio*n* of morbidity**	**Reductio*n* of mortality**	**Improvement of health status relative to clinical outcomes and surrogate outcomes**	**Improvement of health-related quality of life HRQoL**	**Reduction of medication costs**
Professional interventions	Targeted at prescribers	Clinical case analysis and/or evaluation	^*^	^*^	^*^	^*^	^*^	(Hajjar et al., 2007)	(Hajjar et al., 2007)	^*^	^*^	^*^
		Review of drug use	^*^	^*^	^*^	^*^	^*^	(Hajjar et al., 2007)	(Hajjar et al., 2007)	^*^	^*^	^*^
		Educational interventions for prescribers	^*^	^*^	^*^	^*^	^*^	(Hajjar et al., 2007)	(Hajjar et al., 2007)	^*^	^*^	^*^
	Targeted at users	Educational interventions for users and/or caregivers (uniprofessional and/or multiprofessional)	^*^	^*^	^*^	^*^	^*^	(Hajjar et al., 2007)	(Hajjar et al., 2007)	^*^	^*^	^*^
Organizationa interventions	Use of information and communication technology	Active search for user data and information	^*^	^*^	^*^	^*^	^*^	^*^	^*^	^*^	^*^	^*^
		Drug information services	^*^	^*^	^*^	^*^	^*^	^*^	^*^	^*^	^*^	^*^
		Clinical decision-making support systems	^*^	^*^	^*^	^*^	^*^	^*^	^*^	^*^	^*^	^*^
		Use of risk screening tools	^*^	^*^	^*^	^*^	^*^	^*^	^*^	^*^	^*^	^*^
	Provision of pharmaceutical care services		^*^	^*^	^*^	(Lee et al., 2013; Babar et al., 2017)	^*^	^*^	^*^	(Lee et al., 2013)	(Lee et al., 2013; Jórdan-Sánchez et al., 2015)	(Bojke et al., 2010; Jórdan-Sánchez et al., 2015)
	Technical management of medications (medication logistics)		^*^	^*^	^*^	^*^	^*^	^*^	^*^	^*^	(Bojke et al., 2010; Desborougha et al., 2011)	^*^
Financial interventions-incentive programs to change prescribing practices			^*^	^*^	^*^	^*^	^*^	^*^	^*^	^*^	^*^	^*^
Governmental interventions: regulatory interventions - governmental policies that regulate prescriptions			^*^	^*^	^*^	^*^	^*^	^*^	^*^	^*^	^*^	^*^
Multifaceted interventions			^*^	^*^	^*^	^*^	^*^	^*^	^*^	^*^	^*^	^*^

*The references in parentheses indicate the studies that presented evidence for each intervention/outcome studied*.

* *Not studied and/or no evidence*.

*Community-level pharmaceutical interventions to reduce the risks of polypharmacy in the elderly: overview of systematic reviews and economic evaluations*.

### Methodological Quality of Included Studies

The systematic reviews were graded based on the AMSTAR criteria, with scores varying from low to high quality (Supplementary Material 4). The economic evaluation studies were considered of high quality based on the AEES criteria (Supplementary Material 5).

The level of noncompliance with the AMSTAR criteria may be related to the year of publication of the article, as earlier studies tended to have lower scores. The least frequently met AMSTAR criteria, in ascending order, were list of studies (included and excluded), methods for aggregating study results, evaluation, and documentation of scientific quality of included studies, evaluation of probability of publication bias, the inclusion of gray literature, and declaration of conflicts of interest.

### Reported Results

#### Synthesis of Evidence on Interventions for Clinical Outcomes

The results presented in Table 1 show the effects of various categories of interventions (professional, organizational, governmental, and multifaceted) on clinical outcomes related to polypharmacy and medication errors, adherence, and drug-related problems. The reported outcomes include reduced prescription of potentially inappropriate medicines, improved use of appropriate and safe medications, improved adherence, and reduced adverse drug events, adverse drug reactions, drug-drug interactions, and drug-related negative health outcomes.

#### Professional Interventions:

##### Review of drug use

Hajjar et al. (2007), Kaur et al. (2009), Mathumalar et al. (2011), Patterson et al. (2012), Cooper et al. (2015), Jórdan-Sánchez et al. (2015), Olaniyan et al. (2015), Khalil et al. (2017), presented evidence regarding the reduction of polypharmacy, medication errors, prescription of potentially inappropriate medications; improvement of the use of appropriate and safe medications; and improvement of adherence. Hajjar et al. (2007) also found evidence for clinical outcomes related to reduction of drug-related problems, adverse drug events, adverse drug reactions, drug-drug interactions, and drug-related negative health outcomes.

### Clinical Case Analysis and/or Evaluation

Kaur et al. (2009), Alldred et al. (2013), Olaniyan et al. (2015), and have published evidence on clinical outcomes related to the reduction of polypharmacy, medication errors, and prescription of potentially inappropriate medications; improvement of the use of appropriate and safe medications; and improvement of adherence.

#### Educational Interventions Targeted at Prescribers

Kaur et al. (2009), Hajjar et al. (2007), Olaniyan et al. (2015), and Mathumalar et al. (2011) provided evidence for clinical outcomes related to the reduction of polypharmacy, medication errors and prescription of potentially inappropriate medications; improvement of the use of appropriate and safe medications; and improvement of adherence.

#### Educational Interventions Targeted at Users and/or Caregivers

Kaur et al. (2009), Olaniyan et al. (2015), and Mathumalar et al. (2011), and presented evidence for clinical outcomes related to the reduction polypharmacy, medication errors, and prescription of potentially inappropriate medications; improvement of the use of appropriate and safe medications; and improvement of adherence.

### Organizational Interventions

#### Use of Information and/or Communication Technology for Active Search of Data and User Information

Olaniyan et al. (2015) presented evidence for clinical outcomes related to the reduction of polypharmacy, medication errors and prescription of potentially inappropriate medications; improvement of the use of appropriate and safe medications; and improvement of adherence.

#### Use of Information and/or Communication Technology for Drug Information Services

Kaur et al. (2009) presented evidence for clinical outcomes related to the reduction of polypharmacy, medication errors, and prescription of potentially inappropriate medications; improvement of the use of appropriate and safe medications; and improvement of adherence.

#### Use of Information and/or Communication Technology for Clinical Decision-Making Support Systems

Kaur et al. (2009), Mathumalar et al. (2011), and Cooper et al. (2015) presented evidence for clinical outcomes related to the reduction of polypharmacy, medication errors, and prescription of potentially inappropriate medications; improvement of the use of appropriate and safe medications; and improvement of adherence.

#### Use of Information and/or Communication Technology for Risk Screening Tools

Patterson et al. (2012) and Olaniyan et al. (2015) presented evidence for clinical outcomes related to the reduction of polypharmacy, medication errors, and prescription of potentially inappropriate medications; improvement of the use of appropriate and safe medications; and improvement of adherence.

### Provision of Pharmaceutical Care Services

Mathumalar et al. (2011), Patterson et al. (2012), Lee et al. (2013), Sáez-Benito et al. (2013), Cooper et al. (2015), Olaniyan et al. (2015), and Babar et al. (2017) presented evidence for clinical outcomes related to the reduction of polypharmacy, medication errors, and prescription of potentially inappropriate medications; improvement of the use of appropriate and safe medications; and improvement of adherence. Lee et al. (2013) also found data on clinical outcomes regarding the reduction of drug-related problems, adverse drug events, adverse drug reactions, drug-drug interactions, and drug-related negative health outcomes.

### Technical Management of Medications and/or Medication Logistics

Olaniyan et al. (2015) presented evidence for clinical outcomes related to the reduction of polypharmacy, medication errors, and prescription of potentially inappropriate medications; improvement of the use of appropriate and safe medications; and improvement of adherence.

### Governmental Interventions: Regulation of Prescribing Practices

Kaur et al. (2009) presented evidence for clinical outcomes related to the reduction of polypharmacy, medication errors, and prescription of potentially inappropriate medications; improvement of the use of appropriate and safe medications; and improvement of adherence.

### Financial Interventions: Incentive Programs to Change Prescribing Practices

Among the evaluated articles, no article specifically addressed this type of intervention, although there is evidence that such interventions can be effective in improving prescribing quality. It should be noted, however, that the CADTH has published evidence on interventions related to the improvement of prescribing quality using financial interventions (Higgins and Green, 2011).

### Multifaceted Interventions

Hajjar et al. (2007), Alldred et al. (2013), Lee et al. (2013), Cooper et al. (2015), and Olaniyan et al. (2015), and presented evidence for clinical outcomes related to the reduction of polypharmacy, medication errors, and prescription of potentially inappropriate medications; improvement of the use of appropriate and safe medications; and improvement of adherence. Lee et al. (2013) also found data on clinical outcomes related to the reduction of drug-related problems, adverse drug events, adverse drug reactions, drug-drug interactions, and drug-related negative health outcomes.

### Synthesis of Evidence on Epidemiological, Humanistic, and Economic Outcomes and Access to Services

The Table 2 shows the effects of different interventions (professional, organizational, governmental, financial, and multifaceted) on different outcomes: access to services (reduction of outpatient visits, home visits, visits to emergency and emergency services, hospitalizations hospital, time of hospital stay), epidemiological (morbidity and mortality), humanistic (improvement of health status, improvement of health-related quality of life), and economic (reduction of drug costs).

### Professional Interventions:

#### Clinical Case Analysis and/or Evaluation

Hajjar et al. (2007) have presented evidence on epidemiological outcomes including reduction of morbidity and mortality.

#### Review of Drug Use

Hajjar et al. (2007) have presented evidence on epidemiological outcomes including reduction of morbidity and mortality.

#### Educational Interventions Targeted at Prescribers

Hajjar et al. (2007) have presented evidence on epidemiological outcomes including reduction of morbidity and mortality.

#### Educational Interventions Targeted at Users

Hajjar et al. (2007) have presented evidence on epidemiological outcomes including reduction of morbidity and mortality.

### Organizational Interventions:

#### Provision of Pharmaceutical Care Services

Lee et al. (2013) and Babar et al. (2017) presented evidence on the improvement of access to services (output) and reduced hospital admissions. Lee et al. (2013) also found evidence for humanistic outcomes related such improved health status in terms of both clinical and surrogate outcomes. Jórdan-Sánchez et al. (2015) found evidence for improvement of health-related quality of life (HRQoL). Bojke et al. (2010) and Jórdan-Sánchez et al. (2015) found evidence on the reduction of drug costs. There is evidence that, on average, pharmaceutical care is economically viable and cost-effective, with an 80% probability reported by Bojke et al. (2010).

### Equity Considerations in Included Studies

With regard to equity, we found that the criteria described in the studies were limited to place of residence in high-, middle-, and low-income countries. There was no mention of whether individuals lived in urban or rural areas, their race/ethnicity/culture/language or their sex/gender (Table 3). Thus, in general, the included studies did not address equity and did not include subgroup analyses to identify socioeconomic differences.

**Table 3:** PROGRESS framework.

**Article**	**EQUITY: approaches and issues related to equity**							
	**P**	**R**	**O**	**G**	**R**	**E**	**S**	**S**
Khalil et al., 2017	(+)	*	(–)	♀♂	(–)	(–)	(–)	–
Babar et al., 2017	(+)	(–)	(–)	♀♂	(–)	(–)	(–)	–
Loh et al., 2016	(+)	(–)	(–)	♀♂	(–)	(–)	(–)	–
Cooper et al., 2015	(+)	(–)	(–)	♀♂	(–)	(–)	(–)	–
Jokanovic et al., 2015	(+)	(–)	(–)	♀♂	(–)	(–)	(–)	–
Jórdan-Sánchez et al., 2015	(+)	(–)	(–)	♀	(–)	**	***	****
Olaniyan et al., 2015	(+)	(–)	(–)	♀♂	(–)	(–)	(–)	–
Alldred et al., 2013	(+)	(–)	(–)	♀♂	(–)	(–)	(–)	–
Lee et al., 2013	(+)	(–)	(–)	♀♂	(–)	(–)	(–)	–
Sáez-Benito et al., 2013	(–)	(–)	(–)	♀♂	(–)	(–)	(–)	–
Patterson et al., 2012	(+)	*	(–)	♀♂	(–)	(–)	(–)	–
Desborougha et al., 2011	(+)	(–)	(–)	♀♂	(–)	(–)	*****	–
Mathumalar et al., 2011	(+)	(–)	(–)	♀♂	(–)	(–)	(–)	–
Bojke et al., 2010	(+)	(–)	(–)	♀♂	(–)	(–)	(–)	–
Kaur et al., 2009	(–)	(–)	(–)	♀♂	(–)	(–)	(–)	–
Hajjar et al., 2007	(–)	(–)	(–)	♀♂	(–)	(–)	(–)	–

*P, Place of residence; R, Race/ethnicity/culture/language; O, Occupation; G, Sex/gender; R, Religion; E, Education level; S, Socioeconomic status; S, Social capital*.

(+) *, High-, middle- and low-income countries, with no information about whether individuals live in urban or rural areas*.

(–) *, No information*.

♂ *, male; ♀, female*.

* , White and non-white; **, Lack of formal education; ***, Mobility problem; ****, Living with a partner; *****, Own house.

*Source: Pharmaceutical interventions at the community level to reduce risks of polypharmacy in the elderly: an overview of systematic reviews and economic assessments*.

## Discussion and Conclusion

This study aimed to provide an overview of systematic reviews and economic evaluations that addressed community-level pharmaceutical interventions to reduce the risks associated with polypharmacy in the elderly over 65 years. The elderly constitute the age group most at risk of polypharmacy and most susceptible to adverse events. For this population group, care at the community level represents one of the greatest challenges for health systems, especially for universal healthcare systems.

Polypharmacy refers to the prescription of both adequate and inadequate medications. Prescriptions must be made in a way that explicitly considers the overall effects of the total drug regimen and should be based on strong evidence to ensure rational use of medications.

Pharmaceutical care is an important part of universal healthcare systems in regard to ensuring rational use of drugs. Services provided at the points of care that include pharmaceutical care, whether delivered individually or collectively, are particularly important.

## Limitations

This overview used systematic methods and a rigorous approach to identify and provide an up-to-date global synthesis of community-level pharmaceutical interventions that reduce the risks associated with polypharmacy in the elderly over 65 years of age.

A limitation of this study was that the results found did not allow a comparison between the studies, the quality of the evidence presented and the ethical conflicts. It is possible that potentially eligible systematic reviews might have been missed because they used different synonyms of the key descriptors.

The authors of the selected systematic reviews often warned readers to be cautious in the interpretation of the results, especially in view of the difficulty of aggregating data and the heterogeneity of the studies in terms of the variety, types, intensity and multiplicity of indicators and the use of narrative synthesis, as a meta-analysis was not possible.

## Implications for Professional Practice

The categories of interventions included in this overview (professional, organizational, regulatory, financial, and multifaceted) demonstrated the benefits of pharmaceutical care for improving outcomes in the elderly over 65 years of age. There is evidence that an adequate system for managing, prescribing, monitoring, and evaluating the use of medications is effective in reducing polypharmacy and improving adherence to medications, while decreasing drug costs, medication errors, drug-related problems, adverse drug reactions, drug-drug interactions, drug-related negative health outcomes, and hospital admissions. Such systems also improve access to services, the use of safe and adequate medications and health-related quality of life.

### Implications for Research

In terms of implications for research, there is a substantial number of international studies showing that community-level interventions that reduce the risks associated with polypharmacy are complex and varied; there is no single path. However, authors of the systematic reviews selected in this overview highlight issues that remain unanswered, namely:

- Is there a difference between the socioeconomic and cultural profile of the elderly in terms of equity and clinical, humanistic, epidemiological, and economic outcomes?
- What types and/or models of pharmaceutical interventions provide monetary gains when compared to other intervention models?
- Is there a good cost-effectiveness and/or cost-utility ratio (life years gained, disability days avoided, QALY or DALY) in the long term for pharmaceutical care users?
- Are there psychological effects on patients receiving pharmaceutical care in terms of clinical, humanistic, epidemiological, and economic outcomes?
- Is there a positive impact of pharmaceutical care on the cognitive function and functional capacity of elderly patients?
- Which indicators are more specific and sensitive in measuring pharmaceutical interventions and their correlation with clinical, humanistic, epidemiological, and economic outcomes?
- Do multifaceted strategies for pharmaceutical care have a synergistic effect on clinical, humanistic, epidemiological, and economic outcomes?
- What is the minimum time (time scale) and/or frequency (daily, weekly, monthly) of pharmaceutical care provided to elderly patients necessary to be effective and/or efficient?
- Are the positive effects on clinical, humanistic, epidemiological, and economic outcomes persistent in the long-term?

There is a need for further investigation of the effect of various types of pharmaceutical interventions (professional, organizational, regulatory, financial, and multifaceted) on the improvement of pharmaceutical care in the elderly over 65 years of age.

### Implications for Policies and Programs

As for the implications for policies and programs, pharmaceutical care stands out from an economic perspective as it is an efficient intervention to optimize prescribed medications and improve the quality of life in elderly persons taking multiple medications. Results from a cost-utility analysis suggest that pharmaceutical care is cost-effective.

There is evidence—with 80% probability—that pharmaceutical care is economically viable and profitable. This supports its incorporation into pharmaceutical assistance programs and/or policies, especially in universal healthcare systems based on access, quality and rational and sustainable use of medications at all levels of health care.

We know how important it is to ensure the establishment and implementation of evidence-based policies. Thus, we reiterate that, in universal and sustainable healthcare systems, pharmaceutical assistance and/or pharmaceutical care must be based on evidence of the efficacy and safety of the drugs, the effectiveness of the medications and the efficiency of the treatments.

Finally, we recommend that pharmaceutical professionals committed to efficient health policies should be included in multidisciplinary care teams to ensure that the elderly have access to high-quality and safe pharmacotherapy and a better quality of life.

## Author Contributions

OS defined the research questions and prepared the research protocol in conjunction with JB. OS designed the study and conducted the bibliographic searches and the analyses. JB refined the research question in the original draft and contributed to the study design by helping with the literature review and article review. The authors read and approved the final version of the manuscript.

### Conflict of Interest Statement

The authors declare that the research was conducted in the absence of any commercial or financial relationships that could be construed as a potential conflict of interest.

## References

[B1] Alldred D. P. Raynor D. K. Hughes C. Barber N. Chen T. F. Spoor P. (2013). Interventions to optimise prescribing for older people in care homes (Review). Cochrane. Database Syst. Rev. 2:CD009095 10.1002/14651858.CD009095.pub2 23450597

[B2] Babar Z. D. Kousar R. Murtaza G. Azhar S. Khan S. A. Curley L. (2017). Randomized controlled trials covering pharmaceutical care and medicines management: a systematic review of literature. Res. Soc. Admin. Pharm. 1:19 10.1016/j.sapharm.2017.06.008 28651923

[B3] Bojke C. Sculpher M. Campion P. Chrystyn H. Coulton S. Cross B. (2010). Cost-effectiveness of shared Pharmaceutical care for older patients: RESPECT trial findings. Br. J. Gen. Pract. 60:e20–27. 10.3399/bjgp09X482312 20040164 PMC2801802

[B4] Booth A. (2013). PROSPERO's progress and activities 2012/13. Syst. Rev. 2:111. 10.1186/2046-4053-2-111 24330739 PMC3874669

[B5] Brasil Ministério da Saúde (2014). Serviços farmacêuticos na atenção básica à saúde. Secretaria de Ciência, Tecnologia e Insumos Estratégicos. Departamento de Assistência Farmacêutica e Insumos Estratégicos. – Brasília: Ministério da Saúde, 108 p.: il. – (Cuidado farmacêutico na atenção básica; caderno 1).

[B6] Canadian Agency for Drugs Technologies in Health (2018). CADTH Evidence Drive. Search Rx for Change Database. CADTH publishes. Canadian Copyright. Available online at: https://www.cadth.ca/resources/rx-for-change/database/ (accessed August 12, 2018).

[B7] Cooper J. A. Cadogan C. A. Patterson S. M. Kerse N. Bradley M. C. Ryan C.. (2015). Interventions to improve the appropriate use of polypharmacy in older people: a Cochrane systematic review. BMJ Open 5:e009235. 10.1136/bmjopen-2015-009235 26656020 PMC4679890

[B8] Desborougha J. A. Sachb T. Bhattacharya D. Holland R. C. Wright D. J. (2011). A cost-consequences analysis of an adherence focused pharmacist-led medication review servisse. Int. J. Pharmacy Pract. 20, 41–49. 10.1111/j.2042-7174.2011.00161.x 22236179

[B9] Duerden M. Avery T. Payne R. (2013). Polypharmacy and Medicines Optimization. Making it Safe and Sound. London: The King's Fund; First published by The King's Fund Charity Registration Number: 1126980.

[B10] Hajjar E. R. Cafiero A. C. Hanlon J. T. (2007). Polypharmacy in elderly patients. Am. J. Geriatr. Pharmacother. 5, 341–355. 10.1016/j.amjopharm.2007.12.002 18179993

[B11] Higgins J. P. T. Green S. (2011). Cochrane Handbook for Systematic Reviews of Interventions. Vol. 4 *de Wiley Cochrane Series* London: John Wiley & Sons.

[B12] Jokanovic N. Tan E. C. K. Dooley M. J. Kirkpatrick C. M. Bell J. S. (2015). Prevalence and factors associated with polypharmacy in long-term care facilities: a systematic review. JAMDA 16:535.e11. 25869992 10.1016/j.jamda.2015.03.003

[B13] Jórdan-Sánchez F. Malet-Larrea A. Martín J. García-Mochón L. López del Amo M. Martínez-Martínez F. (2015). Cost-utility analysis of a medication review with follow-up service for older adults with polypharmacy in community pharmacies in spain: the conSIGUE Program. PharmacoEconomics 33, 599–610. 10.1007/s40273-015-0270-2 25774017

[B14] Kaur S. Mitchell G. Vitetta L. Roberts M. S. (2009). Interventions that can reduce inappropriate prescribing in the elderly - a systematic review. Drugs Aging. 26, 1013–1028. 10.2165/11318890-000000000-00000 19929029

[B15] Khalil H. Bell B. Chambers H. Sheikh A. Avery A. J. (2017). Professional, structural and organizational interventions in primary care for reducing medication errors. Cochrane Database Syst. Rev. 10:CD003942 10.1002/14651858.CD003942.pub3 28977687 PMC6485628

[B16] Lee J. K. Slack M. K. Martin J. Ehrman C. Chisholm-Burns M. (2013). Geriatric Patient Care by U.S. Pharmacists in healthcare teams: systematic review and meta-analyses. J. Am. Geriatr. Soc. 61, 1119–1127. 10.1111/jgs.12323 23796001

[B17] Leelakanok N. Holcombe A. L. Lund B. C. Gu X. Schweizer M. L. (2017). Association between polypharmacy and death: a systematic review and meta-analysis. J. Am. Pharmac. Assoc. 57, 729–38.e10. 10.1016/j.japh.2017.06.002 28784299

[B18] Loh Z. Cheen M. Wee H. (2016). Humanistic and economic outcomes of pharmacist-provided medication review in the community-dwelling elderly: a systematic review and meta-analysis. J. Clin. Pharmacy Therapeut. 41, 621–633. 10.1111/jcpt.12453 27696540

[B19] Mathumalar L. Shonella S. Dean F. B. Bottle A. Azeem M. (2011). Interventions to optimise prescribing in care homes: systematic review. Age Ageing 40, 150–162. 10.1093/ageing/afq161 21262782

[B20] Mendeley J. A. Thomson M. Coyne R. P. (2017). How and When to Reference. Available online at https://www.howandwhentoreference.com (accessed July 7, 2018).

[B21] Moher D. Liberati A. Tetzlaff J. Altman D. G. (2009). The PRISMA Group. Preferred reporting items for systematic reviews and meta-analyses: the PRISMA statement. PLoS Med. 6:e1000097 10.1371/journal.pmed.1000097 19621072 PMC2707599

[B22] National Collaborating Centre for Methods and Tools (2015). PROGRESS Framework: Applying An Equity Lens to Interventions. Hamilton, ON: McMaster University.

[B23] Olaniyan J. O. Ghaleb M. Dhillon S. Robinson P. (2015). Safety of medication use in primary care. Int. J. Pharmacy Pract. 23, 3–20. 10.1111/ijpp.12120 24954018

[B24] O'Neill J. Tabish H. Welch V. Petticrew M. Pottie K. Clarke M.. (2014). Applying an equity lens to interventions: using, PROGRESS ensures consideration of socially stratifying factors to illuminate inequities in health. J. Clin. Epidemiol. 67, 56–64. 10.1016/j.jclinepi.2013.08.005 24189091

[B25] Ouzzani M. Hammady H. Fedorowicz Z. Elmagarmid A. (2016). Rayyan — a web and mobile app for systematic reviews. Syst. Rev. 5:210. 10.1186/s13643-016-0384-4 27919275 PMC5139140

[B26] Patterson S. M. Hughes C. Kerse N. Cardwell C. R. Bradley M. C. (2012). Interventions to improve the appropriate use of polypharmacy for older people. Cochr. Database Syst. Rev. 5:CD008165. 10.1002/14651858.CD008165.pub2 22592727

[B27] Pinto S. P. L. C. Von-Simson O. R. M. (2012). Instituições de longa permanência para idosos no Brasil: sumário da legislação. Rev. Brasil. Geriatr. Gerontol. 15, 169–174. 10.1590/S1809-98232012000100018

[B28] Sáez-Benito L. Fernandez-Llimos F. Feletto E. Gastelurrutia M. A. Martinez-Martinez F. Benrimo S. (2013). Evidence of the clinical effectiveness of cognitive pharmaceutical services for aged patients. Age Ageing 42, 442–449. 10.1093/ageing/aft045 23676212

[B29] Santos C. M. C. Pimenta C. A. M. Nobre M. C. (2007). A estratégia PICO para a construção da pergunta de pesquisa e busca de evidências. Rev. Latino-Am. Enfermagem 15, 508–511. 10.1590/S0104-11692007000300023

[B30] Shea B. J. Grimshaw J. M. Wells G. A. Boers M. Andersson N. Hamel C.. (2007). Development of AMSTAR: a measurement tool to assess the methodological quality of systematic reviews. BMC Med. Res. Methodol. 7:10. 10.1186/1471-2288-7-10 17302989 PMC1810543

[B31] Silva E. M. Galvão T. F. Pereira M. G. Silva M. T. (2014). Estudos de avaliação econômica de tecnologias em saúde: roteiro para análise crítica. Rev. Panam. Salud. Publica. 35, 219–227. 24793870

[B32] Soler O. Barreto J. (2018). Pharmaceutical Interventions in Community Level to Reduce Risks of Polypharmacy in the Elderly: An Overview of Systematic Reviews and Economic Evaluations. York, UK: PROSPERO: International prospective register of systematic reviews. Available online at: http://www.crd.york.ac.uk/PROSPERO/display_record.php?ID=CRD42018093788 10.3389/fphar.2019.00302 PMC6454558 31001117

[B33] United Nations Department of Economic and Social Affairs, Population Division (2017). World Population Prospects: The 2017 Revision, Key Findings and Advance Tables. Working Paper No. ESA/P/WP/248.

[B34] World Health Organization (2015). World Report on Ageing and Health. Geneva: World Health Organization, 246.

