# Supplementary material for: Community-Level Pharmaceutical Interventions to Reduce the Risks of Polypharmacy in the Elderly: Overview of Systematic Reviews and Economic Evaluations
Source: Front Pharmacol. 2019 Apr 2;10:302. doi: 10.3389/fphar.2019.00302 (PMC6454558; doi:10.3389/fphar.2019.00302)
Supplement: Supplementary file 1 [file Table_1.DOCX]

**SUPPLEMENTARY MATERIAL**

**Community-level pharmaceutical interventions to reduce the risks of polypharmacy in the elderly: overview of systematic reviews and economic evaluations**

Orenzio Soler*1, Jorge Otávio Maia Barreto2.

1 School of Pharmacy. Health Science Institute. Federal University of Pará. Belém. Pará. Brazil.

2 Fiocruz School of Government. Fiocruz Brasília. Osvaldo Cruz Foundation. Brasília. Federal District. Brazil.

* E-mail: [orenziosoler@ufpa.br](mailto:orenziosoler@ufpa.br)

**Supplementary Material 1** | Search strategies

**Supplementary Material 1 |** Search strategies

Review question: Which community-level pharmaceutical interventions reduce the risks associated with polypharmacy in the elderly population over 65 years of age?

- Pharmaceutical intervention: Occurs when the professional performs pharmacotherapeutic monitoring; that is, it is an ongoing process that identifies and solves problems related to medications, performing interventions aimed at increasing effectiveness and reducing the risks of pharmacotherapy (1).
- Polypharmacy: This is the prescription of multiple drugs for an individual. There is still no consensus as to the number of drugs prescribed for both short-term (acute) and long-term (chronic) conditions. Problem polypharmacy is where multiple medications are inappropriately prescribed or where the intended benefit of the medication is not achieved (2).
- Elderly : The elderly is defined as chronological age, so the elderly person is 60 years of age or older in developing countries and 65 years of age or older in developed countries. It is important to recognize that chronological age is not a precise marker for the changes that accompany aging. There are significant differences related to the state of health, participation and levels of independence among people of the same age (3).

In accordance with the PICO guidelines (4), studies with the following characteristics were included: Population (P): Individuals over 65 years of age; Intervention (I): Pharmaceutical interventions (pharmaceutical care); Control (C): No pharmaceutical intervention or any other intervention; and Outcome (O): Clinical, epidemiological, humanistic and economic outcomes.

This overview covered studies published in the following databases: Cochrane Library, Epistemonikos, Health Evidence, Health Systems Evidence, Virtual Health Library (Portuguese acronym: BVS) and Google Scholar. There was no language or time restriction. Systematic reviews, with or without meta-analysis, and economic evaluations were included. The search strategy included medical subject headings (MeSH) and health sciences descriptors (DeCS), using the keywords "Elderly", "Polypharmacy" and "Pharmaceutical intervention". The search was adapted to the various electronic databases.

| **Base** | **Strategy: subject or descriptors** |
| --- | --- |
| Cochrane Library | ("pharmaceutical care" OR pharmaceutical OR pharmacy) AND (elderly OR older OR aged OR "old age" OR elder) |
| Epistemonikos | ("pharmaceutical care" OR pharmaceutical OR pharmacy) AND (elderly OR older OR  aged OR "old age" OR elder) |
| Health Evidence | ("pharmaceutical care" OR pharmaceutical OR pharmacy) AND (elderly OR older OR aged OR "old age" OR elder) |
| Health Systems Evidence | ("pharmaceutical care" OR pharmaceutical OR pharmacy) AND (elderly OR older OR aged OR "old age" OR elder) |
| Virtual Health Library (Portuguese acronym: BVS) | ("pharmaceutical care" OR pharmaceutical OR pharmacy) AND (elderly OR older OR aged OR "old age" OR elder) |
| Google Scholar | ("pharmaceutical care" OR pharmaceutical OR pharmacy) AND (elderly OR older OR aged OR "old age" OR elder) AND (systematic review* OR meta-analyses* OR economic evaluation*) |

Studies focused on other age groups, such as adolescents and adults aged between 18 and 64 years, were excluded, along with studies that addressed interventions at other levels of care. The interventions of interest were those focused on identifying and solving problems related to polypharmacy, pharmaceutical care and reduction of the risks of medication use, at the community level. There was no language or time restriction, , including articles published up to May 2018.

**References**

1. Conselho Federal de Farmácia (CFF). Competências para a atuação clínica do farmacêutico: Relatório do I Encontro Nacional de Educadores em Farmácia Clínica e Matriz de Competências para a Atuação Clínica / Conselho Federal de Farmácia. – Brasília: Conselho Federal de Farmácia, 2017. 124 p.: il. ISBN 978-85-89924-21-4

2. Duerden, Martin; Avery, Tony; Payne, Rupert. Polypharmacy and medicines optimization. Making it safe and sound. Published by The King’s Fund. First published 2013 by The King’s Fund Charity registration number: 1126980. ISBN: 978 1 909029 18 7

3. Organização Mundial da Saúde (OMS). Envelhecimento Ativo: uma Política de saúde. Brasília: Organização Pan-Americana da Saúde, 2005.

4. Santos CMC, Pimenta CAM, Nobre MC. A estratégia PICO para a construção da pergunta de pesquisa e busca de evidências.*Rev. Latino-Am. Enfermagem* [online] (2007) 15(3):508-511. ISSN 1518-8345.  http://dx.doi.org/10.1590/S0104-11692007000300023.
