# Supplementary material for: Community-Level Pharmaceutical Interventions to Reduce the Risks of Polypharmacy in the Elderly: Overview of Systematic Reviews and Economic Evaluations
Source: Front Pharmacol. 2019 Apr 2;10:302. doi: 10.3389/fphar.2019.00302 (PMC6454558; doi:10.3389/fphar.2019.00302)
Supplement: Supplementary file 2 [file Table_2.DOCX]

**SUPPLEMENTARY MATERIAL**

**Community-level pharmaceutical interventions to reduce the risks of polypharmacy in the elderly: overview of systematic reviews and economic evaluations**

Orenzio Soler*1, Jorge Otávio Maia Barreto2.

1 School of Pharmacy. Health Science Institute. Federal University of Pará. Belém. Pará. Brazil.

2 Fiocruz School of Government. Fiocruz Brasília. Osvaldo Cruz Foundation. Brasília. Federal District. Brazil.

* E-mail: [orenziosoler@ufpa.br](mailto:orenziosoler@ufpa.br)

**Supplementary Material 2 |** Terms and definitions related to pharmaceutical care or pharmaceutical services in Brazil

**Pharmacotherapeutic follow-up**: It is the professional practice in which the pharmacist is responsible for the patient's needs related to the medications. This is done by detecting Drug-Related Problems for the prevention and resolution of Drug-Related Negative Health Outcomes. This service implies a commitment and must be provided in a continuous, systematized and documented way, in collaboration with the patient and other professionals of the health system, in order to obtain concrete results that improve the quality of life of the patient (1).

**Adherence to treatment**: The degree of correspondence and agreement of the patient with the recommendations of the physician or other health professional regarding the intake of medications, follow-up of the diet and changes in the habits of life (2).

**Pharmaceutical anamnesis**: Patient data collection procedure performed by the pharmacist through an interview with the purpose of knowing his health history, elaborating the pharmacotherapeutic profile and identifying his health-related needs (3).

**Pharmaceutical Assistance**: It is configured as an important part of the Unified Health System and a fundamental component for actions to promote and improve the conditions of health care for the population, with the purpose of strengthening access strategies and Rational Use of Medicines. That is, a set of actions and services aimed at ensuring comprehensive therapeutic assistance - individual and collective - and the promotion, protection and recovery of health in public and private establishments engaged in pharmaceutical activities (3).

**Medication reconciliation**: Activity described by hospital accreditation manuals in order to increase patient safety. It is a process that consists of obtaining a complete and accurate list of the medications used by the patient and then comparing them with the prescription in all care transitions (admission, hospital discharge or transfer between hospitalization units). When discrepancies are found, they may be considered medication errors (4).

**Pharmaceutical care or pharmaceutical services:** Integrates health education actions, which include ongoing education activities for the health team and general health promotion activities, and actions to promote the rational use of medicines, with the development of assistance and technical-pedagogical activities. The care activity, practiced at the points of care, includes pharmaceutical clinic services, which can be offered to the user individually and / or in shared care with other members of the health team (4).

**Medication error**: It is any avoidable event that, in fact or potentially, can lead to inappropriate use of medication. This means that improper use may or may not harm the patient, and it does not matter whether the medication is under the control of health professionals, the patient or the consumer (5).

**Medication error research**: Evaluate whether a drug is properly treated within the drug management system, which comprises prescribing, transcribing, dispensing, administering stages and monitoring its use (6).

**Adverse drug event**: Is any damage caused by the use of one or more medicinal products for therapeutic purposes, thus covering adverse drug reactions and medication errors (7).

**Pharmacy clinic**: Area of pharmacy focused on science and practice of rational use of medicines, in which pharmacists provide care to patients in order to optimize pharmacotherapy, promote health and well-being and prevent diseases (8).

**Community pharmacy**: This is a service delivery unit to provide pharmaceutical assistance, health care and individual and collective health guidance, in which the handling and / or dispensing of magisterial, officinal, pharmacopoeial or industrialized medicines, cosmetics , pharmaceutical inputs, pharmaceutical products and related (3).

**Hospital pharmacy**: This is a hospital unit that has, among other objectives to ensure the safe and rational use of prescription drugs and respond to the demand for drugs of hospitalized patients. The hospital pharmaceutical care constitutes itself as a complex and relevant in the management of health systems and services system, not only to contemplate one of the basic inputs for patient care, as well as the high costs involved (4).

**Pharmaceutical intervention**: Occurs when the professional performs the pharmacotherapeutic monitoring; that is, it is an ongoing process that identifies and solves problems related to medications, performing interventions aimed at increasing effectiveness and reducing the risks of pharmacotherapy (3).

**Potentially inappropriate medication**: It can be defined as the drug and / or classes of drugs that should be avoided in people 65 years of age or older, when safer alternatives are available; that is, they do not pose unnecessary risks. The term Potentially Inappropriate Prescription encompasses excessive prescription (polypharmacy), prescription and sub-prescription errors. The main challenge for health professionals is to strike a balance between treating diseases and avoiding drug-related harm (9).

**Potentially inappropriate prescription**: It encompasses the use of drugs that produce a significant risk of adverse drug events. Prescription may be considered inadequate when an effective therapy exists to treat the same condition associated with a lower risk (9).

**Polypharmacy or polymedication**: It is the use of several medications concomitantly, and many authors use the term for the use of 5 or more medications within a same period. The more drugs a patient uses, the greater the risk of adverse effects and interactions (10).

**Medication-related problems**: These are situations that in the process of drug use cause or may cause the onset of a negative result associated with medication (11).

**Adverse drug reaction**: Defined as any harmful or unexpected effect occurring after administration of the doses normally used in man for prophylaxis, diagnosis or treatment of a disease or for modification of physiological function (12).

**Drug-related negative health outcomes**: They result in the health of patients not adapted to the objective of pharmacotherapy and associated with the use or failure to use drugs (11).

**Patient safety**: Reduction, to an acceptable minimum, of the risk of unnecessary harm associated with health care (10).

**Rational use of medication or rational use of drugs**: The process by which patients receive medicines appropriate to their clinical needs, in doses appropriate to their individual characteristics, for the appropriate period and at the lowest possible cost, for themselves and for society (5).

**Safe use of medication or safe use of drugs**: No accidental or preventable injury during the use of medicines. Safe use encompasses activities to prevent and minimize the damage caused by adverse events, resulting from the process of using the medicines (9).

**References**

1. Organización Panamericana de la Salud (OPS). Servicios Farmacéuticos basados en la Atención Primaria de Salud: documento de posición de la OPS/OMS. Washington DC: OPS, 2013. 106 p.

2. World Health Organization (WHO). SABATÉ, E. (ed). Adherence to Long-Term Therapies: Evidence for Action. Geneva, Switzerland. 2003.

3. Brasil. Ministério da Saúde. Secretaria de Ciência, Tecnologia e Insumos Estratégicos. Departamento de Assistência Farmacêutica e Insumos Estratégicos. Serviços farmacêuticos na atenção básica à saúde. Brasília: Ministério da Saúde, 2014. 108 p.: il. – (Cuidado farmacêutico na atenção básica; caderno 1). ISBN 978-85-334-2196-7.

4. Brasil. Ministério da Saúde. Secretaria de Ciência, Tecnologia e Insumos Estratégicos. Departamento de Assistência Farmacêutica e Insumos Estratégicos. Capacitação para implantação dos serviços de clínica farmacêutica. Brasília: Ministério da Saúde, 2014. 308 p.: il. (Cuidado farmacêutico na atenção básica; caderno 2) ISBN 978-85-334-2198-1.

5. Word Health Organization (WHO). The rational use of drugs: report of the conference of experts. Nairobi 1985 Jul 25-29. Geneva: WHO; 1987).

6. Aronson JK. Medication errors: what they are, how they happen, and how to avoid them. *Quaterly JMed* 2009; 102:513–521. ISSN:0033-5622 (Print); 0033-5622 (Linking).

7. Word Health Organization (WHO). Conceptual Framework for the International Classification for Patient Safety Version 1.1. More than words. Technical Report January. 2009. WHO/IER/PSP/2010.2).

8. Conselho Federal de Farmácia (CFF). Competências para a atuação clínica do farmacêutico: Relatório do I Encontro Nacional de Educadores em Farmácia Clínica e Matriz de Competências para a Atuação Clínica / Conselho Federal de Farmácia. – Brasília: Conselho Federal de Farmácia, 2017. 124 p.: il. ISBN 978-85-89924-21-4)

9. Word Health Organization (WHO). WHO Global Patient Safety Challenge: Medication Without Harm: WHO’s Third global patient safety challenge. 2017.

10. Word Health Organization (WHO). Conceptual Framework for the International Classification for Patient Safety Version 1.1. More than words. Technical Report January 2009. WHO/IER/PSP/2010.2

11. Universidad de Granada (España): Comité de Consenso. Grupo de Investigación en Atención Farmacéutica (CTS-131); Grupo De Investigación en Farmacología (CTS-164); Universidad de Granada (España); Fundación Pharmaceutical Care España Sociedad Española de Farmacia Comunitaria (SEFaC). Tercer Consenso de Granada sobre Problemas Relacionados con Medicamentos (PRM) y Resultados Negativos asociados a la Medicación (RNM). Ars Pharm 2007; 48 (1): 5-17. ISSN 0004-2729; ISSN: 2340-9894

12. Organização Mundial da Saúde (OMS). A importância da Farmacovigilância. Monitorização da segurança dos medicamentos. Título original: *The importance of pharmacovigilance*. Brasília: Organização Pan-Americana da Saúde, 2005. 48p. ISBN 85-87943-34-0
