# Supplementary material for: Community-Level Pharmaceutical Interventions to Reduce the Risks of Polypharmacy in the Elderly: Overview of Systematic Reviews and Economic Evaluations
Source: Front Pharmacol. 2019 Apr 2;10:302. doi: 10.3389/fphar.2019.00302 (PMC6454558; doi:10.3389/fphar.2019.00302)
Supplement: Supplementary file 3 [file Table_3.DOCX]

**SUPPLEMENTARY MATERIAL**

**Community-level pharmaceutical interventions to reduce the risks of polypharmacy in the elderly: overview of systematic reviews and economic evaluations**

Orenzio Soler*1, Jorge Otávio Maia Barreto2.

1 School of Pharmacy. Health Science Institute. Federal University of Pará. Belém. Pará. Brazil.

2 Fiocruz School of Government. Fiocruz Brasília. Osvaldo Cruz Foundation. Brasília. Federal District. Brazil.

* E-mail: [orenziosoler@ufpa.br](mailto:orenziosoler@ufpa.br)

**Supplementary Material 3 |** List of excluded studies

1. Al Hamid A, Ghaleb M, Aljadhey H, Aslanpour Z. A systematic review of hospitalization resulting from medicine-related problems in adult patients. Br J Clin Pharmacol / 78:2 / 202–217. DOI:10.1111/bcp.12293
2. Avery AJ, Rodgers S, Cantrill JA, Armstrong S, Cresswell K, Eden M, Elliott RA, Howard R, Kendrick D, Morris CJ, Prescott RJ, Swanwick G, Franklin M, Putman K, Boyd M, Sheikh A. A pharmacist-led information technology intervention for medication errors (PINCER): a multicentre, cluster randomised, controlled trial and cost-eff ectiveness analysis Lancet 2012; 379: 1310–19. DOI:10.1016/S0140-6736(11)61817-5
3. Bell S, McLachlan AJ, Aslani P, Whitehead P, Chen TF. Community pharmacy services to optimise the use of medications for mental illness: a systematic review. Australia and New Zealand Health Policy 2005, 2:29 doi: 10.1186/1743-8462-2-29
4. Chhabra PT, Rattinger GB, Dutcher SK, Hare ME, Parsons KL, Zuckerman IH. Medication reconciliation during the transition to and from long-term care settings: A systematic review. Research in Social and Administrative Pharmacy 8 (2012) 60–75
5. Chinthammit C, Armstrong EP, Warholak TL. A Cost-Effectiveness Evaluation of Hospital Discharge Counseling by Pharmacists. Journal of Pharmacy Practice 25(2) 201-208. 2012. DOI: 10.1177/0897190011418512
6. Clyne B, Bradley MC, Hughes C, Fahey T, Lapane KL. Electronic Prescribing and Other Forms of Technology to Reduce Inappropriate Medication Use and Polypharmacy in Older People: A Review of Current Evidence. Clin Geriatr Med 28 (2012) 301–322. doi:10.1016/j.cger.2012.01.009
7. da Silva DT, Santos AP, Aguiar PM, da Silva WB, de Lyra DP Jr. Analysis of research quality regarding pharmaceutical intervention in elderly residents of long-term care facilities: A systematic review.  Journal of the American Geriatrics Society. JULY 2010–VOL. 58, NO. 7. DOI: 10.1111/j.1532-5415.2010.02932.x
8. Gallagher J , O'Sullivan D, McCarthy S, Gillespie P , Woods N, O'Mahony D, Byrne S. Structured Pharmacist Review of Medication in Older Hospitalised Patients: A Cost-Effectiveness. Analysis Drugs and Aging (2016) 33(4) 285-294. DOI 10.1007/s40266-016-0348-3
9. Gellad WF, Grenard JL, Marcum ZA. A Systematic Review of Barriers to Medication Adherence in the Elderly: Looking Beyond Cost and Regimen Complexity. AmJ Geriatr Pharmacother. 2011;9:11–23. doi:10.1016/j.amjopharm.2011.02.004
10. George J, Elliott RA, Stewart DC. A Systematic Review of Interventions to Improve Medication Taking in Elderly Patients Prescribed Multiple Medications. Drugs Aging 2008; 25 (4): 307-324.
11. Higgins N, Regan C. A systematic review of the effectiveness of interventions to help older people adhere to medication regimes. Age and Ageing 2004; 33: 224–229. DOI: 10.1093/ageing/afh072
12. Holland R, Desborough J, Goodyer L, Hall S, Wright D, Loke YK. Does pharmacist-led medication review help to reduce hospital admissions and deaths in older people? A systematic review and meta-analysis. Br J Clin Pharmacol / 65:3 / 303–316 / 303. 2007. DOI:10.1111/j.1365-2125.2007.03071.x
13. Isetts BJ, Schondelmeyer SW, Artz MB, Lenarz LA, Heaton AH, Wadd WB, Brown LM, Cipolle RJ. Clinical and economic outcomes of medication therapy management services: The Minnesota experience. J Am Pharm Assoc. 2008;48:203–211. Doi: 10.1331/JAPhA.2008.07108
14. Isetts BJ, Schondelmeyer SW, Artz MB, Lenarz LA, Heaton AH, Wadd WB, Brown LM, Cipolle RJ. Clinical and economic outcomes of medication therapy management services: The Minnesota experience. J Am Pharm Assoc. 2008;48:203–211. Doi: 10.1331/JAPhA.2008.07108
15. Jano E, Aparasu RR. Healthcare Outcomes Associated with Beers' Criteria: A Systematic Review. TheAnnals of Pharmacotherapy - 2007March, Volume 41. DOI 10.1345/aph.1H473
16. Krumme AA, Isaman DL, Stolpe SF, Dougherty S, Choudhry NK. Prevalence, Effectiveness, and Characteristics of Pharmacy-Based Medication Synchronization Programs. Am J Manag Care. 2016;22(3):179-186
17. Leelakanok N, Holcombe AL, Lund BC, Gu X, Schweizer ML. Association between polypharmacy and death: A systematic review and meta-analysis. Journal of the American Pharmacists Association 57 (2017) 729e738. http://dx.doi.org/10.1016/j.japh.2017.06.002
18. Lundh CMA. Medication review in hospitalised patients to reduce morbidity and mortality. Cochrane Database of Systematic Reviews 2013, Issue 2. Art. No.: CD008986. DOI: 10.1002/14651858.CD008986.pub2.
19. Marcum ZA, Handler SM, Wright R, Hanlon JT. Interventions to Improve Suboptimal Prescribing in Nursing Homes: A Narrative Review. American Journal Geriatric Pharmacotherapy, 8(3), 183–200. https://doi.org/10.1016/j.amjopharm.2010.05.004
20. Meid AD, Lampert A, Burnett A, Seidling HM, Haefeli WE. The impact of Pharmaceutical care interventions for medication underuse in older people: a systematic review and meta-analysis. Br J Clin Pharmacol / 80:4. 2015. DOI:10.1111/bcp.12657
21. Metsälä E, Vaherkoski U. Medication errors in elderly acute care – a systematic review. Scand J Caring Sci; 2014; 28; 12–28. doi: 10.1111/scs.12034
22. Morrison A, Wertheimer AI. Evaluation of studies investigating the effectiveness of pharmacists’ clinical services. Am J Health-Syst Pharm—Vol 58 Apr 1, 2001.
23. Obreli-Neto PR, Marusic S, Guidoni CM, Baldoni Ade O, Renovato RD, Pilger D, Cuman RK, Pereira LR. Economic Evaluation of a Pharmaceutical Care Program for Elderly Diabetic and Hypertensive Patients in Primary Health Care: A 36-Month Randomized Controlled Clinical Trial. Journal of Managed Care & Specialty Pharmacy 2015 vol: 21 (1) pp: 66-75
24. Ojeleye O, Avery A, Gupta  V, Boyd M. The evidence for the effectiveness of safety alerts in electronic patient medication record systems at the point of pharmacy order entry: a systematic review. BMC Medical Informatics and Decision Making 2013, 13:69 https://doi.org/10.1186/1472-6947-13-69
25. Redston MR, Hilmer SN, Mclachlan A, Clough A, Gnjidic D. Prevalence of Potentially Inappropriate Medication Use in Older Inpatients with and without Cognitive Impairment: A Systematic Review. Journal of Alzheimer’s Disease December 2017. Journal of Alzheimer's disease: JAD 61(4):1-14. . DOI:10.3233/JAD-170842
26. Redston MR, Hilmer SN, McLachlan AJ, Clough AJ, Gnjidic D. Prevalence of Potentially Inappropriate Medication Use in Older Inpatients with and without Cognitive Impairment: A Systematic Review. Journal of Alzheimer's Disease (2018) 61(4) 1639-1652. DOI:10.3233/JAD-170842
27. Reeve E, To J, Hendrix I, Shakib S, Roberts MS, Wiese MD. Patient Barriers to and Enablers of Deprescribing: A Systematic Review. Drugs and Aging. 2013.
28. Rollason V, Vogt N. Reduction of polypharmacy in the elderly: a systematic review of the role of the pharmacist. Drugs and Aging 2003; 20(11): 817-832.
29. Schlenk EA, Bernardo LM, Organist LA, Klem ML, Engberg S. Optimizing Medication Adherence in Older Patients: A Systematic. J Clin Outcomes Manag. 2008 December 1; 15(12): 595–606.
30. Smith SM, Wallace E, O'Dowd T, Fortin M. Interventions for improving outcomes in patients with multimorbidity in primary care and community settings (Review). Cochrane Database of Systematic Reviews 2016, Issue 3. Art. No.: CD006560 DOI: 10.1002/14651858.CD006560.pub3.
31. Sturgess IK, McElnay JC, Hughes CM, Crealey G. Community pharmacy-based provision of pharmaceutical care to older patients. Pharm World Sci 2003; 25(5): 218–226.
32. Suggett E, Marriott J. Risk Factors Associated with the Requirement for Pharmaceutical Intervention in the Hospital Setting: A Systematic Review of the Literature. Drugs - Real World Outcomes (2016) 3:241–263. DOI 10.1007/s40801-016-0083-4
33. Thillainadesan J, Gnjidic D, Green S, Hilmer SN. Impact of Deprescribing Interventions in Older Hospitalised Patients on Prescribing and Clinical Outcomes: A Systematic Review of Randomised Trials. Drugs Aging. 2018. https://doi.org/10.1007/s40266-018-0536-4
34. Tjia J, Velten SJ, Parsons C, Valluri S, Briesacher BA. Studies to Reduce Unnecessary Medication Use in Frail Older Adults: A Systematic Review. Drugs & Aging · March 2013. DOI: 10.1007/s40266-013-0064-1
35. Verrue CLR, Petrovic M, Mehuys E, Remon JP, Stichele RV . Pharmacists’ Interventions for Optimization of Medication Use in Nursing Homes. Drugs Aging 2009; 26 (1): 37-49.1170-229X/09/0001-0037/$49.95/0
36. Wimmer BC, Cross AJ, Jokanovic N, Wiese MD, George J, Johnell K, Diug B, Bell JS. Clinical Outcomes Associated with Medication Regimen Complexity in Older People: A Systematic Review. Journal of the American Geriatrics Society (2017) 65(4) 747-753. DOI:10.1111/jgs.14682
37. Yourman L, Concato J, Agostini JV. Use of Computer Decision Support Interventions to Improve Medication Prescribing in Older Adults: A Systematic Review. Am] Geriatr Pharmacother. 2008;6: 119-129. doi: 10.1016/j.amjopharm.2008.06.001.
