# Supplementary material for: Community-Level Pharmaceutical Interventions to Reduce the Risks of Polypharmacy in the Elderly: Overview of Systematic Reviews and Economic Evaluations
Source: Front Pharmacol. 2019 Apr 2;10:302. doi: 10.3389/fphar.2019.00302 (PMC6454558; doi:10.3389/fphar.2019.00302)
Supplement: Supplementary file 4 [file Table_4.DOCX]

**SUPPLEMENTARY MATERIAL**

**Community-level pharmaceutical interventions to reduce the risks of polypharmacy in the elderly: overview of systematic reviews and economic evaluations**

Orenzio Soler*^1^, Jorge Otávio Maia Barreto^2^.

^1^ School of Pharmacy. Health Science Institute. Federal University of Pará. Belém. Pará. Brazil.

^2^ Fiocruz School of Government. Fiocruz Brasília. Osvaldo Cruz Foundation. Brasília. Federal District. Brazil.

* E-mail: [orenziosoler@ufpa.br](mailto:orenziosoler@ufpa.br)

**Supplementary Material 4** | Methodological assessment of systematic reviews

**SUPPLEMENTARY MATERIAL 4** | Methodological assessment of included systematic reviews

|  |  | **AMSTAR*** | | | | | | | | | | | |
| --- | --- | --- | --- | --- | --- | --- | --- | --- | --- | --- | --- | --- | --- |
|  |  | **1** | **2** | **3** | **4** | **5** | **6** | **7** | **8** | **9** | **10** | **11** | **Rating** |
| Khalil et al. 2017 (20) | | Yes | Yes | Yes | Yes | Yes | Yes | Yes | Yes | Yes | Yes | Yes | 11/11 |
| Babar et al. 2017 (21) | | Yes | No | Yes | N/A | No | Yes | Yes | Yes | Yes | Yes | Yes | 9/10 |
| Loh et al. 2016 (22) | | Yes | Yes | Yes | N/A | N/A | Yes | Yes | Yes | Yes | Yes | No | 8/9 |
| Cooper et al. 2015 (23) | | Yes | Yes | Yes | N/A | No | Yes | Yes | Yes | Yes | Yes | Yes | 9/10 |
| Jokanovic et al. 2015 (24) | | Yes | Yes | Yes | N/A | No | Yes | Yes | Yes | Yes | Yes | No | 8/10 |
| Olaniyan et al. 2015 (26) | | Yes | N/A | Yes | No | No | Yes | No | No | N/A | No | Yes | 4/9 |
| Alldred et al. 2013 (27) | | Yes | Yes | Yes | N/A | No | Yes | Yes | Yes | Yes | Yes | Yes | 9/10 |
| Lee et al. 2013 (28) | | Yes | Yes | Yes | N/A | No | Yes | Yes | Yes | Yes | Yes | Yes | 9/10 |
| Sáez-Benito et al (29) | | Yes | Yes | Yes | N/A | No | Yes | Yes | Yes | Yes | Yes | Yes | 9/10 |
| Patterson et al. 2012 (30) | | Yes | Yes | Yes | N/A | Yes | Yes | Yes | Yes | Yes | Yes | Yes | 10/10 |
| Mathumalar et al. 2011 (32) | | Yes | Yes | Yes | No | Yes | Yes | Yes | Yes | No | No | Yes | 8/11 |
| Kaur et al. 2009 (34) | | Yes | N/A | Yes | No | No | Yes | Yes | Yes | Yes | Yes | No | 7/11 |
| Hajjar et al. 2007 (35) | | Yes | No | Yes | No | No | Yes | Yes | Yes | No | No | No | 5/11 |

AMSTAR contains 11-items to appraise the methodological aspects of the systematic reviews. All 11-items were scored as “Yes”, “No”, “Can’t Answer” or “Not Applicable”. AMSTAR comprises the following items: 1. ‘a priori’ design provided; 2. duplicate study selection/data extraction; 3. comprehensive literature search; 4. status of publication as inclusion criteria (i.e., grey or unpublished literature); 5. list of studies included/excluded provided; 6. characteristics of included studies documented; 7. scientific quality assessed and documented; 8. appropriate formulation of conclusions (based on methodological rigor and scientific quality of the studies); 9. appropriate methods of combining studies (homogeneity test, effect model used and sensitivity analysis); 10. assessment of publication bias (graphic and/or statistical test); and 11. conflict of interest statement.

*Adapted from: Shea BJ, Grimshaw JM, Wells GA , Boers M, Andersson N, Hamel C, Porter AC, Tugwell P, Moher D, Bouter LM. Development of AMSTAR: a measurement tool to assess the methodological quality of systematic reviews. BMC Medical Research Methodology (2007) 7(10). https://doi.org/10.1186/1471-2288-7-10
