# Supplementary material for: Community-Level Pharmaceutical Interventions to Reduce the Risks of Polypharmacy in the Elderly: Overview of Systematic Reviews and Economic Evaluations
Source: Front Pharmacol. 2019 Apr 2;10:302. doi: 10.3389/fphar.2019.00302 (PMC6454558; doi:10.3389/fphar.2019.00302)
Supplement: Supplementary file 5 [file Table_5.DOCX]

**SUPPLEMENTARY MATERIAL**

**Community-level pharmaceutical interventions to reduce the risks of polypharmacy in the elderly: overview of systematic reviews and economic evaluations**

Orenzio Soler*^1^, Jorge Otávio Maia Barreto^2^.

^1^ School of Pharmacy. Health Science Institute. Federal University of Pará. Belém. Pará. Brazil.

^2^ Fiocruz School of Government. Fiocruz Brasília. Osvaldo Cruz Foundation. Brasília. Federal District. Brazil.

* E-mail: [orenziosoler@ufpa.br](mailto:orenziosoler@ufpa.br)

**Supplementary Material 5** | Methodological assessment of included economic evaluation studies – AEES*

| **Studies** | **AEES items** | | | | | | | | | | | | | | | | |
| --- | --- | --- | --- | --- | --- | --- | --- | --- | --- | --- | --- | --- | --- | --- | --- | --- | --- |
|  | **1** | **2** | **3** | **4** | **5** | **6** | **7** | **8** | **9** | **10** | **11** | **12** | **13** | **14** | **15** | **16** | **17** |
| Jórdan-Sánchez et al. 2015 (1) | Yes | Yes | Yes | Yes | Yes | Yes | Yes | Yes | Yes | Yes | N/A | N/A | N/A | N/A | Yes | Yes | Yes |
| Desborougha et al. 2011 (2) | Yes | Yes | Yes | Yes | Yes | Yes | Yes | Yes | Yes | Yes | N/A | N/A | N/A | N/A | Yes | Yes | Yes |
| Bojke et al. 2010 (3) | Yes | Yes | Yes | Yes | Yes | Yes | Yes | Yes | Yes | Yes | N/A | N/A | N/A | N/A | Yes | Yes | Yes |
| **Studies** | **AEES** **items** | | | | | | | | | | | | | | | | |
|  | **18** | **19** | **20** | **21** | **22** | **23** | **24** | **25** | **26** | **27** | **28** | **29** | **30** | **31** | **32** | **33** | **Rating** |
| Jórdan-Sánchez et al. 2015 (1) | Yes | N/A | N/A | Yes | Yes | Yes | Yes | Yes | Yes | Yes | Yes | Yes | Yes | Yes | Yes | Yes | **27/27** |
| Desborougha et al. 2011 (2) | Yes | N/A | N/A | Yes | Yes | Yes | Yes | Yes | Yes | Yes | Yes | Yes | Yes | Yes | Yes | Yes | **27/27** |
| Bojke et al. 2010 (3) | Yes | N/A | N/A | Yes | Yes | Yes | Yes | Yes | Yes | Yes | Yes | Yes | Yes | Yes | Yes | Yes | **27/27** |

AEES contains 33-items to appraise the methodological aspects of the economic evaluation studies. All 33-items were scored as “Yes”, “No”, “It is not clear” or “Not Applicable”. AEES comprises the following items: 1. Was the study question adequately, clearly and responsibly; 2. Has the study's target population been clearly described; 3. Were the main alternatives included in the study as well as a comprehensive description of the alternatives analyzed; 4. Was the time horizon of the model long enough to reflect the main differences - cost and outcome in health - among the strategies analyzed; 5. Was the study's perspective informed; 6. Does the study analyze both costs and health outcomes; 7. Has the type of economic evaluation been reported; 9. Have health outcome measures been clearly described and relevant to the study question; 10. Have the sources of estimates of health outcomes been described and justified and are they in line with the target population; 11. Were methods and assumptions used to extrapolate short-term results into final (medium- or long-term) results, which are described and justified; 12. If the estimates of health outcomes come from a clinical trial, does the research protocol reflect what would occur regularly in clinical practice; 13. If estimates of health outcomes have been systematically reviewed, has the quality of evidence been reported; 14. If the estimates of health outcomes come from observational studies or assumptions, was the use of this information due to lack of evidence of better quality; 15. Were the costs clearly stated; 16. Is the measurement of costs in line with the perspective adopted in the study; 17. Has the method used to calculate costs been described and adequate; 18. Was there information about the currency and the period in which the costs were collected; 19. If the costs were collected in different periods, was there an adjustment for inflation; 20. Were future costs and outcomes adjusted for the same discount rate, and was this adequate; 21. Was an analytical model used and is it appropriate to the objectives proposed in the study; 22. Do the states of health represented in the analytical model reflect the biological process of the disease and the consequences of the use of research technologies; 23. Was methodological uncertainty circumvented; 24. Has structural uncertainty been circumvented; 25. Was uncertainty about heterogeneity circumvented; 26. Was the uncertainty about the parameters circumvented; 27. Was the presentation of the study results based on any kind of ratio between costs and health outcomes; 28. Was the discussion of the study results broad enough, including the key aspects relevant to patients and the decision-maker; 29. Was there information about the internal consistency of the model; 30. Was there information about the external consistency of the model; 31. Has the study funding been adequately described; 32. Have the authors stated their potential conflicts of interest; 33. Has the study been approved by any institution that is qualified in research ethics.

*Adapted from: Silva EM, Galvão TF, Pereira MG, Silva MT. Estudos de avaliação econômica de tecnologias em saúde: roteiro para análise crítica. Rev Panam Salud Publica. 2014;35(3):219–27.

**References**

1. Jórdan-Sánchez F, Malet-Larrea A, Martín J, García-Mochón L, López del Amo M, Martínez-Martínez F, Gastelurrutia-Garralda M, García-Cárdenas V, Sabater-Hernández D, Sáez-Benito L, Benrimoj S. Cost-Utility Analysis of a Medication Review with Follow-Up Service for Older Adults with Polypharmacy in Community Pharmacies in Spain: The conSIGUE Program. PharmacoEconomics (2015) 33:599-610. DOI 10.1007/s40273-015-0270-2

2. Desborougha JA, Sachb T, Bhattacharya D, Holland RC, Wright DJ. A cost-consequences analysis of an adherence focused pharmacist-led medication review servisse. International Journal of Pharmacy Practice (2011) 20:41-49. DOI: 10.1111/j.2042-7174.2011.00161.x

3. Bojke C, Sculpher M, Campion P, Chrystyn H, Coulton S, Cross B, Richmond S, Farrin A, Hill G, Hilton A, Miles J, Russell I, Chi KeiWong I. Cost-effectiveness of shared Pharmaceutical care for older patients: RESPECT trial findings. Br J Gen Pract (2010) January: 21-27. DOI: 10.3399/bjgp09X482312.
