# Supplementary material for: Community-Level Pharmaceutical Interventions to Reduce the Risks of Polypharmacy in the Elderly: Overview of Systematic Reviews and Economic Evaluations
Source: Front Pharmacol. 2019 Apr 2;10:302. doi: 10.3389/fphar.2019.00302 (PMC6454558; doi:10.3389/fphar.2019.00302)
Supplement: Supplementary file 6 [file Table_6.DOCX]

**SUPPLEMENTARY MATERIAL**

**Community-level pharmaceutical interventions to reduce the risks of polypharmacy in the elderly: overview of systematic reviews and economic evaluations**

Orenzio Soler*1, Jorge Otávio Maia Barreto2.

1 School of Pharmacy. Health Science Institute. Federal University of Pará. Belém. Pará. Brazil.

2 Fiocruz School of Government. Fiocruz Brasília. Osvaldo Cruz Foundation. Brasília. Federal District. Brazil.

* E-mail: [orenziosoler@ufpa.br](mailto:orenziosoler@ufpa.br)

**Supplementary Material 6 |** Characteristics of included studies

**Supplementary Material 6 | Characteristics of included studies (Continued)**

| **Article** | **Objective** | **Method** | **Limitation** | **Evidence** | **Conclusion** | **Quality^*; **^** |
| --- | --- | --- | --- | --- | --- | --- |
| Khalil et al. 2017  (1) | To determine the effectiveness of professional, organizational, and structural interventions compared to standard care to reduce medication errors leading to hospital admissions, urgency and emergency department visits, and mortality. | Cochrane Systematic Review of Randomized Trials published until 2013.  Descriptive statistics. Meta-analysis. | Size of samples.  Probability of bias.  Difficulty in aggregating data. | Interventions at the primary health care level to reduce avoidable Medication Errors did not provide significant differences in the number of hospital admissions or in the time of hospitalizations, urgency and emergency department visits, or mortality. | Overall, the interventions included in this review demonstrated benefits of pharmaceutical interventions on the reduction of inappropriate drugs for polymedicated elderly. | AMSTAR 11/11 |
| Babar et al. 2017  (2) | To analyze and describe the evidence of the clinical effectiveness of Pharmaceutical Care. | Overview of Systematic Reviews of Randomized Controlled Clinical Trials and Meta-analyzes, published by 2012. Narrative synthesis. | Quality of evidence.  Multiplicity of indicators. | It has been found in most studies that interventions in the field of Pharmaceutical Care have led to significant improvements in clinical outcomes and / or hospitalizations. | Different types of Pharmaceutical Interventions reduce the number of drugs and improve the adequacy of prescriptions. | AMSTAR 9/10 |
| Loh et al. 2016  (3) | To evaluate the effects of the Medication Use Review on Health-Related Quality of Life (HRQL) and health costs. | Systematic Review of Randomized Controlled Clinical Trials published by 2015. Descriptive statistics. Meta-analysis. | Studies drawings. Size of samples.  Probability of bias.  Quality of evidence. | The Drug Use Review did not significantly improve HRQoL and health care costs when compared to usual care. | The humanistic and economic outcomes obtained from the Pharmacist's Revision of Drug Use were like those of the usual treatment. | AMSTAR 9/10 |
| Cooper et al. 2015  (4) | Investigate the effectiveness of interventions aimed at improving the appropriate use of medicines. | Systematic Review of Randomized Controlled Clinical Trials published until 2013. Narrative Synthesis. | Size of samples.  Difficulty in aggregating data.  Probability of bias.  Quality of evidence. | Interventions have demonstrated improvements in appropriate polypharmacy based on inadequate prescription reductions. However, it remains unclear whether interventions have resulted in significant improvements in clinical outcomes. | The interventions studied demonstrated improvements in appropriate polypharmacy based on the reductions of inadequate prescription. However, it remains unclear whether interventions result in significant clinical improvements. | AMSTAR 9/10 |

**Supplementary Material 6 | Characteristics of included studies (Continued)**

| **Article** | **Objective** | **Method** | **Limitation** | **Evidence** | **Conclusion** | **Quality** |
| --- | --- | --- | --- | --- | --- | --- |
| Jokanovic et al. 2015  (5) | To investigate the prevalence and factors associated with polypharmacy in nursing homes. | Systematic Review of Randomized Controlled Clinical Trials published until 2014. Narrative synthesis. | Heterogeneity of the concept of polypharmacy.  Probability of bias.  Quality of evidence. | The prevalence of polypharmacy in long-term care institutions is high, varying widely between facilities, geographic locations and definitions used. There are associations between polypharmacy and comorbidities; as well as the number of prescribers and discharge. However, cognitive impairment of the elderly, disability in activities of daily living and length of stay in institutions of long-term care are inversely associated with polypharmacy. | There was an association of polypharmacy with comorbidities, recent hospital discharge and number of prescribers. However, there is no association of polypharmacy with elderly cognitive impairment, difficulties in daily activities and length of stay in nursing homes. | AMSTAR 8/10 |
| Jórdan-Sánchez et al. 2015  (6) | To estimate the Cost-Effectiveness Incremental Ratio (ICER) of Drug Use Revision in polymedicated elderly, by means of pharmacotherapeutic follow-up in Spanish community pharmacies compared to those who normally have their medication dispensed. | Randomized controlled and clustered clinical trial carried out over 6 months in 2014 in 178 community pharmacies in Spain, with the target population being polymedicated as the target population. Economic Evaluation Studies. | Non-random selection of the elderly sample.  Selection bias.  Ethical conflicts.  Potential contamination problems between groups.  The cost of outpatient treatment and physician visits were not included as they were not available. | Pharmaceutical Care in community pharmacies is an efficient intervention to optimize prescribed medication and improve the quality of life of polymedicated elderly. The results of the cost-utility analysis suggest that Pharmaceutical Care is cost-effective. | Pharmaceutical Care is an effective intervention to optimize prescribed medication and improve the quality of life in the elderly with polypharmacy in community pharmacies. The results of the cost-utility analysis suggest that Pharmaceutical Care is cost-effective. | AEES 27/27 |
| Olaniyan et al. 2015  (7) | Review studies that address medication error rates and identify studies on interventions to prevent medication errors in Primary Health Care. | Systematic Review of Randomized Controlled Clinical Trials published until 2012. Narrative synthesis. | Studies drawings. Probability of bias.  Quality of evidence. | Pharmaceutical Care aimed at susceptible population groups; as well as monitoring the most critical aspects of the prescription process and the use of medications can be effective for the prevention of prescription errors. Existing interventions can offer time-and cost-saving options for drug safety and to ensure better health outcomes. | A Medication Management and Monitoring System, especially to the susceptible populations, directed to the most critical aspects of the process of prescription and use of medicines can be effective for the prevention of prescription errors. | AMSTAR 4/9 |

**Supplementary Material 6 | Characteristics of included studies (Continued)**

| **Article** | **Objective** | **Method** | **Limitation** | **Evidence** | **Conclusion** | **Quality** |
| --- | --- | --- | --- | --- | --- | --- |
| Alldred et al. 2013  (8) | To determine the effect of interventions to optimize prescribing among nursing home residents. | Cochrane Systematic Review of Randomized Trials, published by 2012.  Narrative synthesis. | Studies drawings. Probability of bias.  Quality of evidence. | Strategies for drug adequacy can be improved through multifaceted interventions involving review of medication by pharmacists, provision of information on medications, and multidisciplinary clinical case studies. However, it is not clear that these interventions translate into improvements in the outcomes related to adverse drug events, hospitalizations, mortality and quality of life. The effect of interventions on medication costs was also unclear. | Inappropriate prescription is prevalent in the context of Nursing Homes. Drug-related problems were resolved through multifaceted interventions involving pharmacy medication review, information technology, and multidisciplinary clinical case studies. There is no evidence on how this translates into improvements in clinical outcomes; humanistic and economic. | AMSTAR 9/10 |
| Lee et al. 2013  (9) | Investigate the effects of Pharmaceutical Care on patient-oriented geriatric outcomes in the United States of America. | Systematic Review of Clinical Randomized Controlled Trials and Meta-Analysis, published by 2012 Meta-analysis. | Studies drawings.  Size of samples.  Probability of bias.  Quality of evidence. | Pharmaceutical Care has significantly greater benefit over other types of usual care in all fields of research; that is, they were consistent for the improvement of therapeutic results, adherence, safety and hospitalization. | Pharmaceutical intervention has favorable effects on outcomes in therapeutics, safety, hospitalization and adherence. Pharmacists should be inserted into the care teams for the elderly. | AMSTAR 9/10 |
| Sáez-Benito et al. 2013  (10) | To analyze and describe the evidence of the clinical effectiveness of Pharmaceutical Care in elderly patients. | Systematic Review of Randomized Controlled Clinical Trials and Meta-analyzes, published by 2011. Meta-analysis. | Studies drawings.  Size of samples.  Probability of bias.  Quality of evidence. | Pharmaceutical Care is an effective strategy to reduce the number of drugs, improve drug suitability and adherence to treatment. However, no correlation was reported between interventions and hospitalization, mortality, and improvement of the functional capacity of the patient. | Through Pharmaceutical Care, different types of interventions reduce the number of medications and improve the adequacy of prescriptions; thereby reducing Drug Related Problems. | AMSTAR 9/10 |

**Supplementary Material 6 | Characteristics of included studies (Continued)**

| **Article** | **Objective** | **Method** | **Limitation** | **Evidence** | **Conclusion** | **Quality** |
| --- | --- | --- | --- | --- | --- | --- |
| Patterson et al. 2012  (11) | To determine which interventions, alone or in combination, are effective in improving the appropriate use of polypharmacy and the reduction of drug-related problems in elderly patients. | Cochrane Systematic Review of Randomized Controlled Trials and Meta-analyzes, published by 2010. Metanalysis. | Heterogeneity regarding variation, types, intensity and  duration of interventions, or differences in follow-up time.  Probability of bias.  Quality of evidence. | Pharmaceutical Care, through multidisciplinary actions, seems to improve the appropriate polypharmacy, being beneficial for the reduction of inadequate prescription and Problems Related to Medications; as well as in encouraging the appropriate use of medicines and promoting health education. However, there are uncertainties about the effect of these interventions on hospital admissions and adverse drug events. | Pharmaceutical Care, through multidisciplinary interventions, seems to improve appropriate polypharmacy. | AMSTAR 10/10 |
| Desborougha et al. 2011  (12) | Cost-Consequence Analysis. | Randomized Clinical Trial in the Norfolk Medicines Support Service, a cost-benefit analysis of Pharmaceutical Care, through Pharmacy-led Drug Use Review in patients not adhering to treatment. Year 2010. Economic Evaluation Studies. | Lack of group control and failure to reach sample size. | Costs of Pharmaceutical Care through the Drug Use Review were offset by reduced hospital admissions for emergencies and reduced cost of medication. There was a significant increase in Drug Adhesion, with the Health-Related Quality of Life standard significantly maintained over the 6-month follow-up. | There was a significant increase in the report of Drug Adhesion. There was no significant reduction in HRQOL over the 6-month follow-up. | AEES 27/27 |
| Mathumalar et al. 2011  (13) | Systematically review the effects of interventions to optimize prescribing in health care homes. | Systematic Review of Randomized and Non-Randomized Controlled Trials published by 2010 Narrative synthesis. | The categorization of articles was not always straightforward.  Studies drawings. Probability of bias.  Quality of evidence. | There is substantial evidence that pharmaceutical interventions are effective and promising to improve prescriptions in nursing homes; for multi-professional interventions related to preschool and caregiver education work through the use of information technology. | This review has demonstrated distinct results with substantial evidence and promising options for some of the interventions. Workers' Education, Computer Decision Support Systems, and Reviews of Pharmaceutical Use of Medications have demonstrated improvements in the profile of appropriate prescriptions. | AMSTAR 8/11 |

**Supplementary Material 6 | Characteristics of included studies (Last)**

| **Article** | **Objective** | **Method** | **Limitation** | **Evidence** | **Conclusion** | **Quality** |
| --- | --- | --- | --- | --- | --- | --- |
| Bojke et al. 2010  (14) | To evaluate the cost-effectiveness of Pharmaceutical Care for the elderly compared to usual care, according to the National Institute of Health and Cases of Excellence (NICE) Clinical Reference Standards. | Cost-effectiveness study of Pharmaceutical Care in relation to usual care for people aged > 75 years who lived in the nursing home and who receive prescriptions of five or more medications. Year 2008. Economic Evaluation Studies. | Sampling and time limit.  Study design. Probability of bias.  Quality of evidence. | Pharmaceutical Care generates extra modest cost and health benefits compared to usual care. The average incremental cost per QALY gained suggests that Pharmaceutical Care can be cost effective. There is evidence that the Pharmaceutical Care is 80% likely to be economically viable and cost-effective. | RESPECT was the first study to evaluate the cost-effectiveness of community pharmacy care for elderly patients in the UK. There is evidence that, on average, pharmaceutical care is economically viable and cost-effective, with 80% probability. | AEES 27/27 |
| Kaur et al. 2009  (15) | Identify interventions and strategies that can significantly reduce inappropriate prescriptions in the elderly. | Systematic Review of Randomized and Non-Randomized Controlled Trials published by 2008 Narrative Synthesis. | Studies drawings. Probability of bias.  Quality of evidence. | Different interventions have positively improved the prescription pattern, especially: regulatory policies, revision of the use of medicines by pharmacists, educational interventions; computerized decision support systems and the support of multidisciplinary teams for geriatric medicine approaches. | Educational Intervention, Computer System Support, Pharmaceutical Intervention, Geriatric Medicine Service, Multidisciplinary Team, Regulatory Policies, and Multifaceted Approaches have shown that inappropriate or suboptimal drug use has declined over time. | AMSTAR 7/11 |
| Hajjar et al. 2007  (16) | Provide a description of observational studies that examine the epidemiology of polypharmacy and review randomized controlled studies that have been published in the last two decades and designed to reduce polypharmacy in the elderly. | Systematic Review of Randomized Controlled Clinical Trials, published until 2008 Narrative synthesis. | Studies drawings. Probability of bias.  Quality of evidence. | The Drug Use Review, Drug Information and Medical Education provide a reduction in polypharmacy; consequently, improving the evaluated outcomes: Adherence; Inadequate prescribing; Adverse Drug Reactions; Geriatric Syndromes; Morbidity; Mortality. | All interventions demonstrated an improvement in polypharmacy; consequently, on the evaluated outcomes: adherence, inadequate prescription, adverse drug reactions, geriatric syndromes, morbidity and mortality. | AMSTAR 5/11 |

* AMSTAR: A MeaSurement Tool to Assess the Methodological Quality of Systematic Reviews. Adapted from: Shea BJ, Grimshaw JM, Wells GA , Boers M, Andersson N, Hamel C, Porter AC, Tugwell P, Moher D, Bouter LM. Development of AMSTAR: a measurement tool to assess the methodological quality of systematic reviews. BMC Medical Research Methodology (2007) 7(10). https://doi.org/10.1186/1471-2288-7-10

** AEES: Assessment of Economic Evaluation Studies. Adapted from: Silva EM, Galvão TF, Pereira MG, Silva MT. Estudos de avaliação econômica de tecnologias em saúde: roteiro para análise crítica. Rev Panam Salud Publica. 2014;35(3):219–27.

**References**

1. Khalil H, Bell B, Chambers H, Sheikh A, Avery AJ. Professional, structural and organizational interventions in primary care for reducing medication errors. Cochrane Database of Systematic Reviews (2017) 10. Art. No.: CD003942. DOI: 10.1002/14651858.CD003942.pub3

2. Babar ZD, Kousar R, Murtaza G, Azhar S, Khan SA, Curley L. Randomized controlled trials covering pharmaceutical care and medicines management: A systematic review of literature. Research in Social and Administrative Pharmacy xxx (2017) 1:19. http://dx.doi.org/10.1016/j.sapharm.2017.06.008

3. Loh Z, Cheen M, Wee H. Humanistic and economic outcomes of pharmacist-provided medication review in the community-dwelling elderly: A systematic review and meta-analysis. Journal of Clinical Pharmacy and Therapeutics (2016) 41(6):621-633. Doi: 10.1111/jcpt.12453

4. Cooper JA, Cadogan CA, Patterson SM, Kerse N, Bradley MC, Ryan C, Hughes CM. Interventions to improve the appropriate use of polypharmacy in older people: a Cochrane systematic review. BMJ Open (2015) 5:e009235. doi:10.1136/bmjopen-2015-009235

5. Jokanovic N, Tan ECK, Dooley MJ, Kirkpatrick CM, Bell JS. Prevalence and Factors Associated with Polypharmacy in Long-Term Care Facilities: A Systematic Review. JAMDA (2015) 16:535.e11. http://dx.doi.org/10.1016/j.jamda.2015.03.003

6. Jórdan-Sánchez F, Malet-Larrea A, Martín J, García-Mochón L, López del Amo M, Martínez-Martínez F, Gastelurrutia-Garralda M, García-Cárdenas V, Sabater-Hernández D, Sáez-Benito L, Benrimoj S. Cost-Utility Analysis of a Medication Review with Follow-Up Service for Older Adults with Polypharmacy in Community Pharmacies in Spain: The conSIGUE Program. PharmacoEconomics (2015) 33:599-610. DOI 10.1007/s40273-015-0270-2

7. Olaniyan JO, Ghaleb M, Dhillon S, Robinson P. Safety of medication use in primary care. International Journal of Pharmacy Practice (2015) 23:3-20. DOI: 10.1111/ijpp.12120

8. Alldred DP, Raynor DK, Hughes C, Barber N, Chen TF, Spoor P. Interventions to optimise prescribing for older people in care homes (Review). Cochrane Database of Systematic Reviews (2013) 2. Art. nº: CD009095. DOI: 10.1002/14651858.CD009095.pub2.

9. Lee JK, Slack MK, Martin J, Ehrman C, Chisholm-Burns M. Geriatric Patient Care by U.S. Pharmacists in Healthcare Teams: Systematic Review and Meta-Analyses. Journal of the American Geriatrics Society (2013) 61(7):1119-1127. DOI:10.1111/jgs.12323

10. Sáez-Benito L, Fernandez-Llimos F, Feletto E, Gastelurrutia MA, Martinez-Martinez F, Benrimo S. Evidence of the clinical effectiveness of cognitive pharmaceutical services for aged patients. Age and Ageing (2013) 42:442-449. DOI: 10.1093/ageing/aft045

11. Patterson SM, Hughes C, Kerse N, Cardwell CR, Bradley MC. Interventions to improve the appropriate use of polypharmacy for older people. Cochrane Database of Systematic Reviews (2012) 5. Art. nº: CD008165. DOI: 10.1002/14651858.CD008165.pub2.

12. Desborougha JA, Sachb T, Bhattacharya D, Holland RC, Wright DJ. A cost-consequences analysis of an adherence focused pharmacist-led medication review servisse. International Journal of Pharmacy Practice (2011) 20:41-49. DOI: 10.1111/j.2042-7174.2011.00161.x

13. Mathumalar L, Shonella S, Dean FB, Bottle A, Azeem M. Interventions to optimise prescribing in care homes: systematic review. Age and Ageing (2011) 40:150–162 doi: 10.1093/ageing/afq161

14. Bojke C, Sculpher M, Campion P, Chrystyn H, Coulton S, Cross B, Richmond S, Farrin A, Hill G, Hilton A, Miles J, Russell I, Chi KeiWong I. Cost-effectiveness of shared Pharmaceutical care for older patients: RESPECT trial findings. Br J Gen Pract (2010) January: 21-27. DOI: 10.3399/bjgp09X482312.

15. Kaur S, Mitchell G, Vitetta L, Roberts MS. Interventions that can Reduce Inappropriate Prescribing in the Elderly - A Systematic Review. Drugs Aging (2009) 26(12):1013-1028 DOI: 1170-229X/09/0012-1013/$49.95/0

16. Hajjar ER, Cafiero AC, Hanlon JT. Polypharmacy in Elderly Patients. The American Journal of Geriatric Pharmacotherapy (2007) 5(4):341-355. doi:10,1016/j,amj opharm,2007,12,002
