# Supplementary material for: Community-Level Pharmaceutical Interventions to Reduce the Risks of Polypharmacy in the Elderly: Overview of Systematic Reviews and Economic Evaluations
Source: Front Pharmacol. 2019 Apr 2;10:302. doi: 10.3389/fphar.2019.00302 (PMC6454558; doi:10.3389/fphar.2019.00302)
Supplement: Supplementary file 7 [file Table_7.DOCX]

**SUPPLEMENTARY MATERIAL**

**Community-level pharmaceutical interventions to reduce the risks of polypharmacy in the elderly: overview of systematic reviews and economic evaluations**

Orenzio Soler*1, Jorge Otávio Maia Barreto2.

1 School of Pharmacy. Health Science Institute. Federal University of Pará. Belém. Pará. Brazil.

2 Fiocruz School of Government. Fiocruz Brasília. Osvaldo Cruz Foundation. Brasília. Federal District. Brazil.

* E-mail: [orenziosoler@ufpa.br](mailto:orenziosoler@ufpa.br)

**Supplementary Material 7 |** Distribution of studies by country and continent

**Supplementary Material 7 |** Distribution of studies by country and continent

| **References** | **America** | **Europe** | **Asia** | **Africa** | **Oceania** |
| --- | --- | --- | --- | --- | --- |
| Khalil et al. 2017 (1); Babar et al. 2017 (2); Loh et al. 2016 (3); Cooper et al. 2015 (4); Jokanovic et al. 2015 (5); Jórdan-Sánchez et al. 2015 (6); Olaniyan et al. 2015 (7); Alldred et al. 2013 (8); Lee et al. 2013 (9) Sáez-Benito et al. 2013 (10) Patterson et al. 2012 (11) Desborougha et al. 2011 (12); Mathumalar et al. 2011 (13) Bojke et al. 2010 (14) Kaur et al. 2009 (15) Hajjar et al. 2007 (16). | Brazil (4), Canada (21), Colombia (1),  United States of America (USA) (126). | Belgium (8), Czech Republic (12), Denmark (8), Finland (6), France (4), Germany (12),  Italy (13), Northern Ireland (2), Norway (5), Poland (1), Portugal (4), Republic of Ireland (11), Scotland (1),  Spain (10), Sweden (9), Switzerland (5),  The Netherlands (14), United Kingdom (UK) (54). | China (5),  India (1),  Malaysia (4),  Singapore 1),  Taiwan (2),  Thailand (1),  Turkey (1). | Bahrain (2),  Egypt (1),  Iraq (1),  Israel (11),  Jordan (1),  Saudi Arabia (1),  Sudan (1),  United Arab Emirates (UAE) (1). | Australia (36),  New Zealand (2). |
| Total | 151 | 179 | 15 | 19 | 38 |

Note: The same article is likely to be in more than one systematic review.

**References**

1. Khalil H, Bell B, Chambers H, Sheikh A, Avery AJ. Professional, structural and organizational interventions in primary care for reducing medication erros. Cochrane Database of Systematic Reviews (2017) 10. Art. No.: CD003942. DOI: 10.1002/14651858.CD003942.pub3

2. Babar ZD, Kousar R, Murtaza G, Azhar S, Khan SA, Curley L. Randomized controlled trials covering pharmaceutical care and medicines management: A systematic review of literature. Research in Social and Administrative Pharmacy xxx (2017) 1:19. http://dx.doi.org/10.1016/j.sapharm.2017.06.008

3. Loh Z, Cheen M, Wee H. Humanistic and economic outcomes of pharmacist-provided medication review in the community-dwelling elderly: A systematic review and meta-analysis. Journal of Clinical Pharmacy and Therapeutics (2016) 41(6):621-633. Doi: 10.1111/jcpt.12453

4. Cooper JA, Cadogan CA, Patterson SM, Kerse N, Bradley MC, Ryan C, Hughes CM. Interventions to improve the appropriate use of polypharmacy in older people: a Cochrane systematic review. BMJ Open (2015) 5:e009235. doi:10.1136/bmjopen-2015-009235

5. Jokanovic N, Tan ECK, Dooley MJ, Kirkpatrick CM, Bell JS. Prevalence and Factors Associated with Polypharmacy in Long-Term Care Facilities: A Systematic Review. JAMDA (2015) 16:535.e11. http://dx.doi.org/10.1016/j.jamda.2015.03.003

6. Jórdan-Sánchez F, Malet-Larrea A, Martín J, García-Mochón L, López del Amo M, Martínez-Martínez F, Gastelurrutia-Garralda M, García-Cárdenas V, Sabater-Hernández D, Sáez-Benito L, Benrimoj S. Cost-Utility Analysis of a Medication Review with Follow-Up Service for Older Adults with Polypharmacy in Community Pharmacies in Spain: The conSIGUE Program. PharmacoEconomics (2015) 33:599-610. DOI 10.1007/s40273-015-0270-2

7. Olaniyan JO, Ghaleb M, Dhillon S, Robinson P. Safety of medication use in primary care. International Journal of Pharmacy Practice (2015) 23:3-20. DOI: 10.1111/ijpp.12120

8. Alldred DP, Raynor DK, Hughes C, Barber N, Chen TF, Spoor P. Interventions to optimise prescribing for older people in care homes (Review). Cochrane Database of Systematic Reviews (2013) 2. Art. nº: CD009095. DOI: 10.1002/14651858.CD009095.pub2.

9. Lee JK, Slack MK, Martin J, Ehrman C, Chisholm-Burns M. Geriatric Patient Care by U.S. Pharmacists in Healthcare Teams: Systematic Review and Meta-Analyses. Journal of the American Geriatrics Society (2013) 61(7):1119-1127. DOI:10.1111/jgs.12323

10. Sáez-Benito L, Fernandez-Llimos F, Feletto E, Gastelurrutia MA, Martinez-Martinez F, Benrimo S. Evidence of the clinical effectiveness of cognitive pharmaceutical services for aged patients. Age and Ageing (2013) 42:442-449. DOI: 10.1093/ageing/aft045

11. Patterson SM, Hughes C, Kerse N, Cardwell CR, Bradley MC. Interventions to improve the appropriate use of polypharmacy for older people. Cochrane Database of Systematic Reviews (2012) 5. Art. nº: CD008165. DOI: 10.1002/14651858.CD008165.pub2.

12. Desborougha JA, Sachb T, Bhattacharya D, Holland RC, Wright DJ. A cost-consequences analysis of an adherence focused pharmacist-led medication review servisse. International Journal of Pharmacy Practice (2011) 20:41-49. DOI: 10.1111/j.2042-7174.2011.00161.x

13. Mathumalar L, Shonella S, Dean FB, Bottle A, Azeem M. Interventions to optimise prescribing in care homes: systematic review. Age and Ageing (2011) 40:150–162 doi: 10.1093/ageing/afq161

14. Bojke C, Sculpher M, Campion P, Chrystyn H, Coulton S, Cross B, Richmond S, Farrin A, Hill G, Hilton A, Miles J, Russell I, Chi KeiWong I. Cost-effectiveness of shared Pharmaceutical care for older patients: RESPECT trial findings. Br J Gen Pract (2010) January: 21-27. DOI: 10.3399/bjgp09X482312.

15. Kaur S, Mitchell G, Vitetta L, Roberts MS. Interventions that can Reduce Inappropriate Prescribing in the Elderly - A Systematic Review. Drugs Aging (2009) 26(12):1013-1028 DOI: 1170-229X/09/0012-1013/$49.95/0

16. Hajjar ER, Cafiero AC, Hanlon JT. Polypharmacy in Elderly Patients. The American Journal of Geriatric Pharmacotherapy (2007) 5(4):341-355. doi:10,1016/j,amj opharm,2007,12,002
